# Micelles self-assembled by 3-*O*-β-d-glucopyranosyl latycodigenin enhance cell membrane permeability, promote antibiotic pulmonary targeting and improve anti-infective efficacy

**DOI:** 10.1186/s12951-020-00699-y

**Published:** 2020-10-02

**Authors:** Man Zhang, Lili Ye, Hao Huang, Dandan Cheng, Kaixin Liu, Wenbo Wu, Fukui Shen, Zhihong Jiang, Yuanyuan Hou, Gang Bai

**Affiliations:** 1 State Key Laboratory of Medicinal Chemical Biology, College of Pharmacy and Tianjin Key Laboratory of Molecular Drug Research, Nankai University, Haihe Education Park, 38 Tongyan Road, Tianjin, 300353 People’s Republic of China; 2 State Key Laboratory of Quality Research in Chinese Medicine, Macau University of Science and Technology, Taipa, Macau China; 3 Tianjin University of Traditional Chinese Medicine, Tianjin, 300193 People’s Republic of China

**Keywords:** 3-*O*-β-d-glucopyranosyl latycodigenin, Self-assembled nanomicelles, Cell membrane permeability, Lung-targeting therapy

## Abstract

**Background:**

Nanoparticle-based pulmonary drug delivery systems are commonly developed and applied for drug-targeted delivery. They exhibit significant advantages compared to traditional pulmonary drug delivery systems. However, developing the formulation of each drug is a time-consuming and laborious task.

**Results:**

In this study, a universal lung-targeting nanoparticle was designed and constructed. The self-assembled micelles were composed of a platycodon secondary saponin, 3-*O*-β-d-glucopyranosyl platycodigenin 682 (GP-682), based on its specific amphiphilic structure. The GP-682 micelles exhibited a relatively stable zeta potential with a particle size between 60 and 90 nm, and the critical micelle concentration (CMC) value was approximately 42.3 μg/mL. Preincubation of GP-682 micelles markedly enhanced their cell membrane permeability and improved drug uptake in vitro. The results were visualized using fluorescent dye tracing, transmission electron microscopy (TEM) observations and the lactate dehydrogenase (LDH) release assay. The obtained benefits enhanced the distribution of levofloxacin (Lev) in mouse lung tissue and reduced antibiotics overdosing. The acute lung injury mouse model induced by the *Pseudomonas aeruginosa* PA 14 strain demonstrated that preinjection of GP-682 micelles before antibiotic administration resulted in a higher survival rate and anti-infective efficacy in vivo. It also caused reductions in pulmonary injury, bacterial invasion and cytokine expression compared with treatment with Lev alone.

**Conclusions:**

GP-682 micelles are another nanoparticle-based pulmonary drug delivery system and provide a new lung-targeting therapy option.

## Background

As the predominant end-organ of local disease treatment or the administration route for systemic therapies, lungs are a common target of drug delivery [1]. A pulmonary drug delivery system (PDDS) delivers drugs to the lung to produce a local or systemic therapeutic effect. The lung presents unique advantages based on unique physiological features, such as a large specific surface and minimal thickness for drug absorption and permeability [2]. Compared with conventional oral administration, the reduced metabolic response of the lung decreases the degradation of the drug, avoiding the first-pass effect of the liver [3]. PDDS also improves patient compliance compared with injection administration [4]. Peptides and other sensitive drugs are suitable for PDDS. Conventional pulmonary dosage forms primarily consist of pressurized metered-dose inhalers (pMDI), dry powder inhalers (DPI) and spray. Conventional pulmonary preparations have some shortcomings, including a relatively short duration of action, frequent dosing, and poor patient compliance [5]. Therefore, it is necessary to exploit the new drug delivery system of PDDS to overcome the disadvantages of conventional pulmonary preparations.

Nanoparticle-based PDDS has developed rapidly, with examples such as polymer nanoparticles, liposomes, solid lipid nanoparticles (SLN), nanostructured lipid carrier (NLC) and micellar systems [6]. Previous studies indicated that nanoparticle-based PDDS improved the uptake and retention of drugs in the lungs and promoted slow or controlled drug release. Pulmonary administration is affected by various factors, such as particle deposition, lung defense mechanisms, modes of drug administration, drug delivery devices and dosage forms [7, 8]. The existing nanoparticle-based lung delivery systems provide many valuable benefits in pulmonary drug administration. However, some challenges must be addressed. Off-target effects of the drug may induce lung inflammation, pulmonary edema and other diseases. The existing nanoparticle-based lung delivery systems were mostly designed and applied for specific diseases and drugs, making the drug preparation process overly tedious.

Platycodi radix (Jiegeng) is the dried root of *Platycodon grandiflorum* A. DC. (Campanulaceae), and it produces a wide range of pharmacological effects. Jiegeng is widely used to treat respiratory infections. Natural products derived from Jiegeng regulate the production and secretion of airway mucin, explaining the expectorant and antitussive effects of Jiegeng [9]. More than one hundred compounds of saponins, flavones, phenolic acids, polyenes and sterols were identified from Jiegeng [10], with the platycosides considered as the key active ingredients [11]. Modern pharmacological studies showed that platycosides improved cardiovascular system activities and produced neuroprotective, antitumor and antiviral effects [12]. Platycosides also exhibit anti-lipid peroxidation activity, protect liver fibrosis activities, and promote the recovery from liver damage [13]. They also inhibit micro synthesis to relieve hepatitis [14]. Previous studies suggested that some platycodins could self-assemble in aqueous solutions to form nanomicelles using the dissipative particle dynamics simulation method. These results indicate that saponins have great application potential in solubilization [15]. In vivo, metabolic studies showed that the total saponins of Jiegeng were broken down in the gastrointestinal tract. GP-682 and the platycodon secondary saponin 3-*O*-β-d-glucopyranosylplatyconic acid 696 (GPA-696) were the main metabolites [16]. GP-682 had better biological activity than GPA-696 in our previous study [17], which helped increase drug delivery to the lung tissue.

To design a universal lung-targeting nanoparticle and reveal the mechanism of the lung targeting of its adjuvant drugs, PDDS based on GP-682 micelles was developed in this paper. The effects of self-assembled GP-682 micelles on improving cell membrane permeability and increasing cellular uptake were investigated thoroughly. The newly proposed system was then verified with a combination of antibiotics in bacterial infection-induced acute lung injury mice. Compared to treatment with levofloxacin (Lev) alone, preinjection of GP-682 micelles resulted in a greater lung tissue distribution of Lev and a significantly improved anti-infective efficacy.

## Materials and methods

### Materials

The platycodon secondary saponins (PSS) GP-682 and GPA-696 used in this work were synthesized in our laboratory. *N*,*N*-dimethylformamide (DMF) was purchased from Concord Technology Co., Ltd. (Tianjin, China). Nile Red, 9-diethylamino-5H-benzo[alpha]phenoxazine-5-one, was purchased from Aladdin (Beijing, China). FITC was purchased from MedChemExpress (New Jersey, USA). The Cytotoxicity Detection Kit (LDH Activity) was purchased from Roche (Basel, Switzerland). Glutaraldehyde (2.5%) was purchased from GenMed Scientific Inc. (MA, USA). Lev, one of the broad-spectrum antibiotics of quinolone, and terazosin hydrochloride were purchased from Shanghaiyuanye Bio-Technology Co., Ltd. (Shanghai, China). The *Pseudomonas aeruginosa* PA 14 strain was obtained from associate professor Bai Fang of Nankai University (Tianjin, China). A *Pseudomonas aeruginosa* antibody (1001/214) [Alexa Fluor® 488] was purchased from Bio-Techne China Co. Ltd. (Shanghai, China). The concentrations of human IL-6, IL-8 and TNF-α were detected using ELISA kits according to the manufacturer's instructions (Lanpai, Shanghai, China). All cell culture reagents were purchased from Gibco BRL Life Technologies (NY, USA).

### Separation and purification of PSS

The PSS of GP-682 and GPA-696 were isolated and purified from a total saponin extract of platycodi radix [16]. The preparation process was based on our previously published paper [17]. The purified PSS were identified using HPLC and NMR methods. The detailed data are shown in Additional file 1: Fig. S1–S9.

### Preparation and characterization of PSS micelles

GP-682 (0.05–4.00 mg) and GPA-696 (0.20 mg) were dissolved in 400 μL of DMF solvent and diluted to 0.125 mg/mL to 10 mg/mL solutions. The solution was slowly added into 2 mL of pure water under ultrasonic conditions (200 W, SB-25-12DT ultrasonic oscillator, Ningbo Xinzhi Biotechnology Co., Ltd.). After DMF was removed via dialysis against 5Χ 250 mL water for 12 h, the micelle solution was filtered through a 0.45 μm microporous filter membrane (MCE syringe filter) to obtain a self-assembled micellar solution of PSS. The solution was collected, placed within a − 80 °C refrigerator for 30 min to freeze, then placed on a freeze dryer (FZ-1C-50, Beijing Boyikang Experimental Instrument Co., Ltd.) for 24 h at 0 °C to obtain the solid PSS micelles. A Zetasizer (Nano ZS, Malvern Co. Ltd, UK) was used to analyze the particle size, size distribution and the zeta potential of GP-682 micelles scattered in d.d. H_2_O. GP-682 micelles were dropped into a copper mesh (Beijing Zhongjingkeyi Technology Co., Ltd., 200 mesh common carbon support membrane) with hydrophilic treatment. Uranium acetate was used to dye the sample. After air-drying at room temperature, the morphology of GP-682 micelles was observed using TEM with an accelerating voltage of 120 kV (Talos F200C, FEI, USA).

The stability of GP-682 micelles in 10% FBS, water and PBS was evaluated using dynamic light scattering (DLS). GP-682 micelles were dispersed in the three solutions for different time durations, then the stability of GP-682 micelles was detected using DLS. Fluorescence spectrophotometry was applied to determine the CMC value of GP-682 micelles with Nile Red as a probe [18]. GP-682 micelle solutions at concentrations from 0.1 to 1000 μg were prepared. Nile Red (10 μL) in tetrahydrofuran was added to 1 mL of GP-682 micelle solutions. The final concentration of Nile Red was 10^−6^ mol/L. After sonication for 30 min, the fluorescence emission spectra were measured at 560–700 nm with an excitation wavelength of 550 nm. Emission intensity at 633 nm was plotted against the log of GP-682 concentration.

### Cell culture

Human normal lung epithelial cells (BEAS-2B cells) were purchased from the American Type Culture Collection (Rockville, MD) and cultured in RPMI medium 1640 containing 10% FBS, 4.5 g/L glucose, L-glutamine and sodium pyruvate. The cells were cultured at 37 °C with 5% CO_2_ in a humidified incubator. When cells achieved approximately 80% confluence, they were used for the following experiments.

### Investigation of membrane permeability

BEAS-2B cells were cultured in small confocal dishes (NEST, 801001). Different concentrations of GP-682 micelles (10, 50, 100 μg/mL) or GPA-696 micelles (100 μg/mL) were cocultured with cells for 30 min in the culture medium at 37 °C. The FITC (1 × 10^−6^ mol/L) was then added to cells for 10 min at 37 °C. After washing with precooled PBS, the cells were fixed with 4% paraformaldehyde, and 200 μL of a 4′,6-diamidino-2-phenylindole (DAPI) solution was added to the cells for 10 min. Finally, a confocal microscope (Leica TCS SP8) was used to investigate the entry of FITC into the cells. The excitation wavelength was 488 nm, and the emission wavelength ranged from 600 to 670 nm. The fluorescence intensity of FITC was detected by Image J software.

GP-682 micelles (10, 50, 100 μg/mL) diluted in the culture medium were used to pretreat cells for 30 min at 37 °C, then 1 × 10^−6^ mol/L FITC was added for the next 30 min. After the administration, 500 μL trypsin was added to cells for digestion. The trypsin was discarded, the cells were suspended in PBS buffer, the cell suspension was centrifuged at 800 r/min for 3 min, the supernatant was discarded, and 1 mL PBS was added to resuspend the cells. A 40 μm nylon cell sieve was used to filter the cell suspension into a flow sample tube, and 10^4^ cells were collected to obtain the intensity data with a fluorescence flow cytometer (BD FACSCalibur System). The excitation wavelength was 490 nm, and the emission wavelength was 530 nm. FCS Express V3 software was used to analyze the results of the flow cytometry experimentation.

### Transepithelial electrical resistance (TEER)

The BEAS-2B cells were transferred to a cell culture plate (Corning, 3460) with a transwell chamber at a density of 2.5 × 10^4^/cm^2^. RPMI 1640 medium (500 μL) was added to the upper chamber, and 1000 μL was added to the lower chamber. RPMI 1640 medium with 10% FBS was added to cultured cells 24 h later to make the cell replication state consistent. 100 μg/mL GP-682 micelles were added to cells to incubate for 90 min or removed at 30 min. TEER at a different time was detected by an EVOM2 Transmembrane cell resistance meter (World Precision Instruments Inc., Florida, USA). The standardized resistance of the transwell chamber equation is sTER = TER × S.

### Morphological observations using TEM

GP-682 micelles (100 μg/mL) were added to cells at 37 °C for 30 min. BEAS-2B cells without any treatment were used as the control group. The cells were collected and digested with trypsin, and then centrifuged at 1,000 rpm for 10 min. After discarding the supernatant, the cells were washed with precooled normal saline. Cells were then fixed with 2% (v/v) precooled glutaraldehyde and 1% osmium tetroxide. Cells dehydrated with ethanol solutions were embedded with LR white and used for preparing ultrathin sections. The cells were desiccated to the critical point and shadowed with platinum. A transmission electron microscope (TEM) (Hitachi HT7700, Japan) was used to observe the cells.

### Endocytic pathway analysis

To check the endocytosis mediated uptake, BEAS-2B cells were seeded in small confocal dishes and pretreated with different endocytosis inhibitors, including sucrose (clathrin-mediated uptake, 1 μM), methyl-β cyclodextrin (caveolae-mediated uptake, 1 μM) and amiloride (micropinocytosis, 1 μM) in serum-free DMEM for 1 h. To visualize the distribution of GP-682 micelles, Cy5.5 labeled GP-682 was synthesized to prepare GP-682-Cy5.5 micelles. The detailed synthesis process of GP-682-Cy5.5 and the preparation of GP-682-Cy5.5 micelles are shown in Additional file 1: Fig. S10–S13. DAPI was added to cells for 10 min at room temperature. A confocal microscope (Leica TCS SP8) was used to investigate the entry of GP-682-Cy5.5 micelles through endocytosis by the cells. The excitation wavelength was 633 nm, and the emission wavelength was between 653 and 700 nm.

### Lactate Dehydrogenase (LDH) release assay

2 × 10^4^ BEAS-2B cells were seeded in RPMI 1640 medium with 10% FBS for 48 h, and a series of GP-682 micelles (10 to 500 μg/mL) were added for 30 min, or fixed-dose GP-682 micelles (50 μg/mL) were added for a range of time durations (30 to 90 min) in RPMI 1640 medium without FBS. The supernatant (100 μL) was collected and incubated with a mixture of diaphorase/NAD ^+^ and iodotetrazolium chloride (100 μL) for the LDH release detection. After 1 h incubation, the absorbance was tested at 500 nm (Spark 10 M, TECAN, CH). Triton X-100 was added to cells to obtain the maximum release of LDH (Hc) as a positive control. The LDH release of cells without any treatment was set up as a low level-control (Lc). Percentage with regard to control was expressed as: [(treated mean—Lc)/(Hc—Lc)] × 100.

### Preparation and assay of GP-682/Nile Red micelles

The GP-682/Nile Red micelles were prepared using the same ultrasound method as for GP-682 micelle preparation. The proportion of GP-682 to Nile Red was 10:1. Different forms of Nile Red were produced, including Nile Red dissolved in 0.1% DMSO and GP-682/Nile Red micelles. The final concentrations of Nile Red added to cells were identical (1 μg/mL). BEAS-2B cells were cultured in small confocal dishes and divided into three groups: Nile Red group, GP-682/Nile Red micelles group and GP-682 micelles + Nile Red group. In the Nile Red and GP-682/Nile Red micelles groups, the cells were treated with Nile Red or GP-682/Nile Red micelles only. In the GP-682 micelles + Nile Red group, cells were treated with 100 μg/mL GP-682 micelles for 30 min in advance, and the same dose of Nile Red (1 μg/mL) was added. A confocal microscope (Leica TCS SP8) was used to investigate the entry of Nile Red into the cells. The excitation wavelength was 561 nm, and the fluorescence emission spectra were measured at 580–700 nm.

### Distribution analysis of lung tissue using HPLC

Ten male Kunming mice (18–22 g) of SPF grade were purchased from Beijing Vital River Laboratory Animal Technology Co., Ltd. After 1 week of regular rearing, the mice fasted for 12 h before the experiment. The mice were randomly divided into two groups: the Lev administration group (80 mg/kg), and the GP-682 micelles (5 mg/kg) preadministration for 30 min before Lev administration (80 mg/kg) group. The administration method was intraperitoneal injection. The mice were sacrificed at 0.08, 0.25, 0.5, 1.0, 1.5 and 2 h after Lev injection. The lung tissue of the mice was separated after rinsing with normal saline via heart perfusion. One gram of the lung tissue was homogenized with 3 g of a normal saline buffer. After centrifugation at 3,000 rpm for 10 min, 100 μL of the supernatant was added to 100 μL of an internal standard solution (50 μg/mL terazosin hydrochloride methanol solution), followed by 200 μL of methanol. The solution was mixed thoroughly by vortexing and centrifuged at 10,000 rpm for 15 min. The supernatant (320 μL) was removed and blow-dried with nitrogen. The residue was reconstituted with 100 μL of methanol and centrifuged at 10,000 rpm for 15 min. The content of Lev-L in the supernatant was determined using high-performance liquid chromatography (HPLC). The HPLC method was performed with a Shimadzu HPLC (lc-20a) coupled with a fluorescence detector (RF-20A, Shimadzu, Japan). The following chromatographic conditions were used: Phenomenex Luna C18 column (150 mm × 4.6 mm, 5 μm); 10 mmol/L phosphate buffer mobile phase (containing 0.01% triethylamine, pH 3)-acetonitrile (82:18); 1 mL/min flow rate; 295 nm excitation wavelength, 490 nm emission wavelength; 35 °C column temperature; and 20 μL injection volume. GraphPad Prism 8 was used for data processing and analyses. The methodological investigation is detailed in Additional file 1: Figs. S14, S15, Tables S1–S3.

### Acute lung injury model

Male KM mice (18–22 g) were purchased from Beijing Vital River Laboratory Animal Technology Co., Ltd. The mice were housed under standard specific pathogen-free conditions with 12/12-h light/dark cycles at 23 ± 2 °C and free access to water and food. A total of 105 mice were randomly divided into seven groups (15 mice per group): Model group (Mod); GP-682 micelles group (5 mg/kg GP-682 micelles); Lev administration groups (Lev-H, 52 mg/kg Lev; Lev-M, 26 mg/kg Lev; Lev-L, 13 mg/kg Lev); and GP-682 micelles preadministration for 30 min groups (GP-682 micelles + Lev-M, 5 mg/kg GP-682 micelles + 26 mg/kg Lev; GP-682 micelles + Lev-L, 5 mg/kg GP-682 micelles + 13 mg/kg Lev). Mice were anesthetized via an intraperitoneal injection of a 4% chloral solution (4 μL/g). Activated *P. aeruginosa* PA 14 bacteria (1 × 10^8^/20 μL in PBS) were dropped into the nasal cavity to induce an acute lung infection. The mice were immediately given an antibiotic intervention, except in the Model and GP-682 micelles groups. Survival was recorded 6, 8, 10, 12, 14, 16, 18, 20, 22 and 24 h after challenge with the PA-14 bacteria.

Another 48 mice were divided into eight groups, as described in the survival experiment, except that one control group (Con) was added. A mild infection model was used to investigate the effect of combination therapy. Activated PA 14 bacteria were used at 1 × 10^7^/20 μL in PBS for nasal cavity infection. Mice were anesthetized 24 h later via inhalation of ether. Bronchoalveolar lavage (right lung) was performed via the administration of 1 mL of 0.9% saline through a tracheal cannula, and the fluid was collected for cytokine assays. The left lung tissues were eviscerated and fixed in a formaldehyde solution (10%) for hematoxylin–eosin (H&E) staining and bacterial immunofluorescence detection.

### Statistical analysis

The results were reported as the mean values ± SD. Analysis of multiple groups was performed using analysis of variance (one-way ANOVA), and significant differences between two groups were assessed using *t*-tests. The log-rank test was used to analyze the significant differences in the survival rate experiments. Differences of p < 0.05 were considered statistically significant.

## Results and discussion

### Characterization of GP-682 micelles

**Scheme 1.**
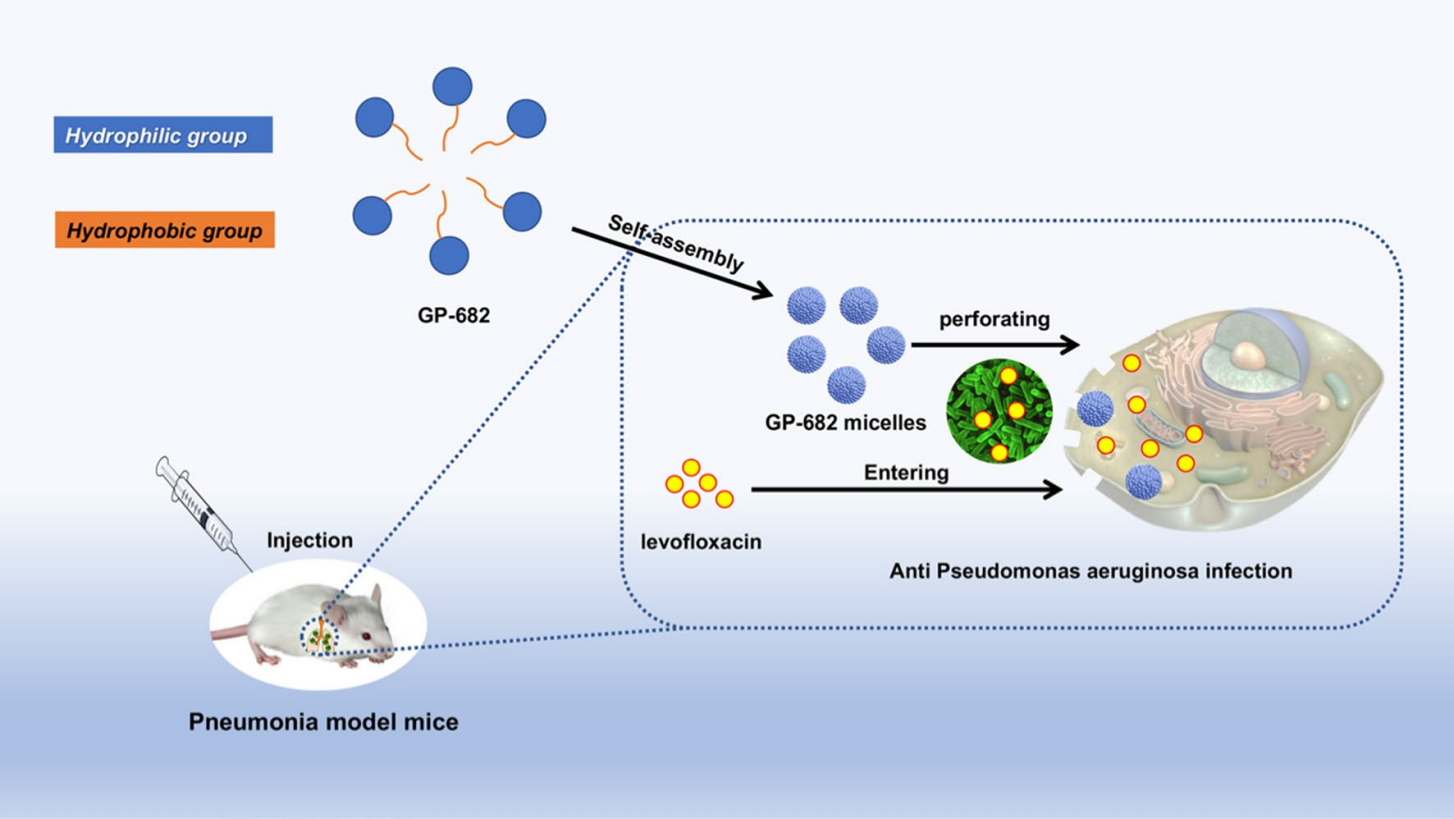
Self-assembled process and application of GP-682 micelles

**Fig. 1:**
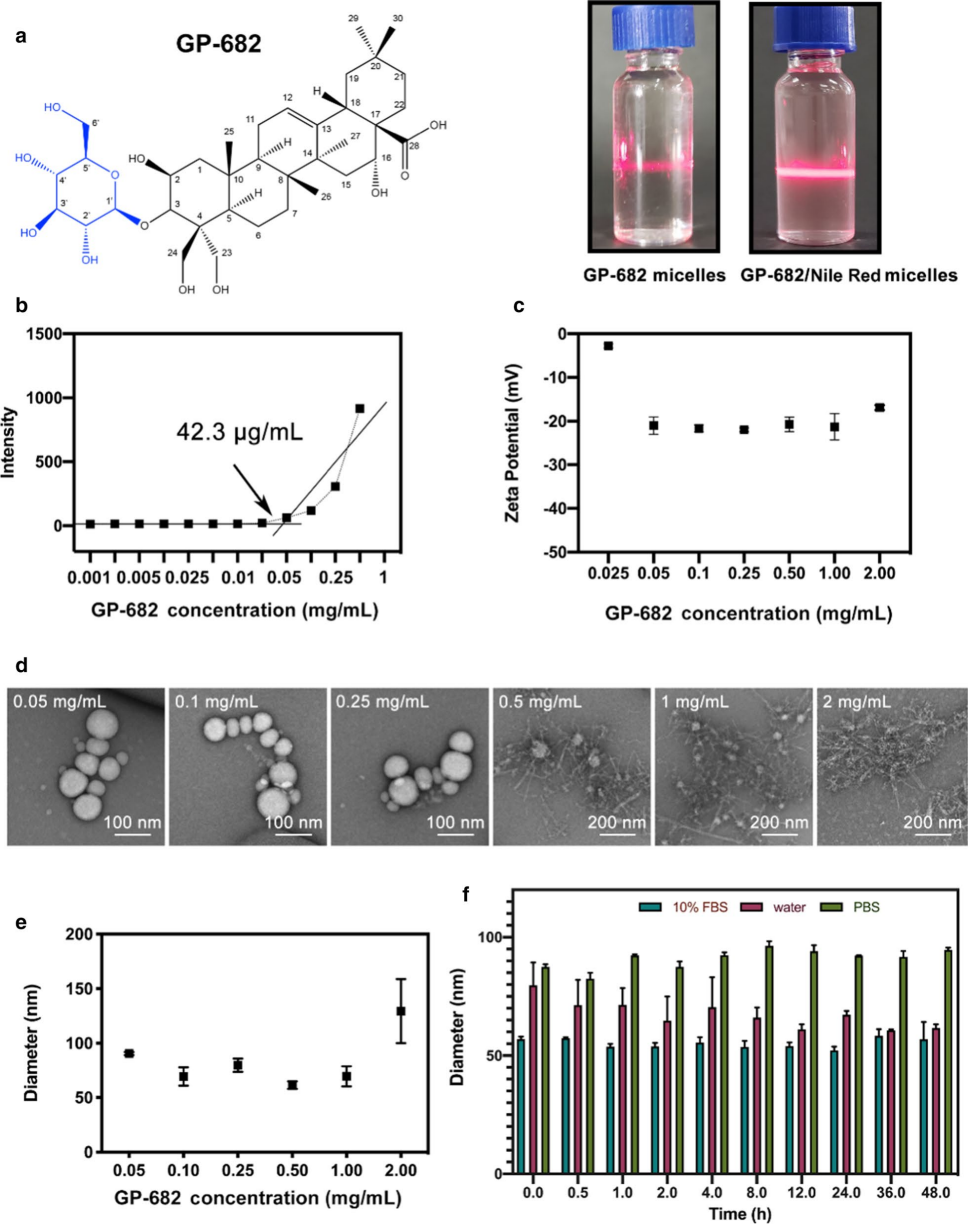
Characterization of GP-682 micelles. **a** The chemical structure of GP-682 and the Tyndall phenomenon of GP-682 micelles and GP-682/Nile Red micelles. **b** The CMC investigation of GP-682. **c** The zeta potential determination of GP-682. **d** The TEM images of GP-682 micelles. **e** The particle size distribution of GP-682 micelles in different concentrations detected using DLS. **f** Stability of mixed GP-682 micelles in 10% FBS, water and PBS. (Data are presented as means ± SD, n = 3)

Saponins are amphiphilic compounds composed of one or more hydrophilic sugars and one lipophilic steroid or triterpenic moiety. Due to their amphiphilic structure, saponins can self-aggregate and interact specifically with membrane lipids [19]. This research was implemented based on this characteristic of saponins. The self-assembly process and application of GP-682 micelles are shown in Scheme 1. The amphiphilic GP-682 was identified as a key PSS (shown in Fig. 1a (left)), which was reported to improve the dosimetry of [^18^F]-phillygenin in lung tissue [17]. The preparations of GP-682 micelles and GP-682/Nile Red micelles were performed using their self-aggregation capacity. The Tyndall phenomenon (shown in Fig. 1a (right)) suggested that the micelles were formed. The CMC value is a very important parameter of micelles. It involves the amphiphilic copolymer’s self-assembly ability and influences the structural stability of micelles in vitro and in vivo [20]. Therefore, the CMC value of GP-682 micelles was measured using Nile Red as a hydrophobic fluorescence probe. As shown in Fig. 1b, the CMC of GP-682 was approximately 42.3 μg/mL. The CMC value of saponin also exerts a significant impact on its permeabilization ability [21]. The relatively low CMC value ensures the stability of GP-682 micelles in the extracellular matrix and benefits its permeability of cell membranes compared with other saponins. Unlike conventional nanomaterials, saponins are relatively safe and effective adjuvants to enhance the absorption of drugs [15]. Compared with other saponins isolated from the roots of Jiegeng, which showed an alternative hemolytic effect in vitro, especially platycodin D, the GP-682 micelles would be safer and more innocuous [22]. BEAS-2B cells cocultured with GP-682 micelles for 24 h remained healthy as detected by the Cell Counting Kit-8 cytotoxicity test and cell cycle detection, which demonstrated that GP-682 micelles were safe as used here (Additional file 1: Figs. S16, S17).

The zeta potential is also an important index to characterize the stability of colloidal dispersions. The zeta potential of GP-682 micelles in different concentrations was characterized. As shown in Fig. 1c, the zeta potential of most micelles was negatively charged at nearly − 20 mV, except for preparation concentrations less than 0.05 mg/mL. Therefore, the preparation concentration of GP-682 was limited from 0.05 to 2 mg/mL in subsequent experiments. The TEM images of GP-682 micelles are exhibited in Fig. 1d. GP-682 micelles prepared between 0.05 and 0.25 mg/mL exhibited a narrow size distribution and appeared spherical with homogeneous morphology. The optimum size distribution and particle size of GP-682 micelles were characterized between 60 to 90 nm, similar to the TEM measurement and suitable for cellular uptake experiments and injection into mice. To detect GP-682 micelle stability in 10% FBS, water and PBS, the respective size distributions for different incubation times were detected using DLS. The results demonstrated that the particle size of GP-682 micelles did not change significantly during 48 h in the three conditions (Fig. 1f).

### Preincubation of GP-682 micelles improved cellular uptake

**Fig. 2:**
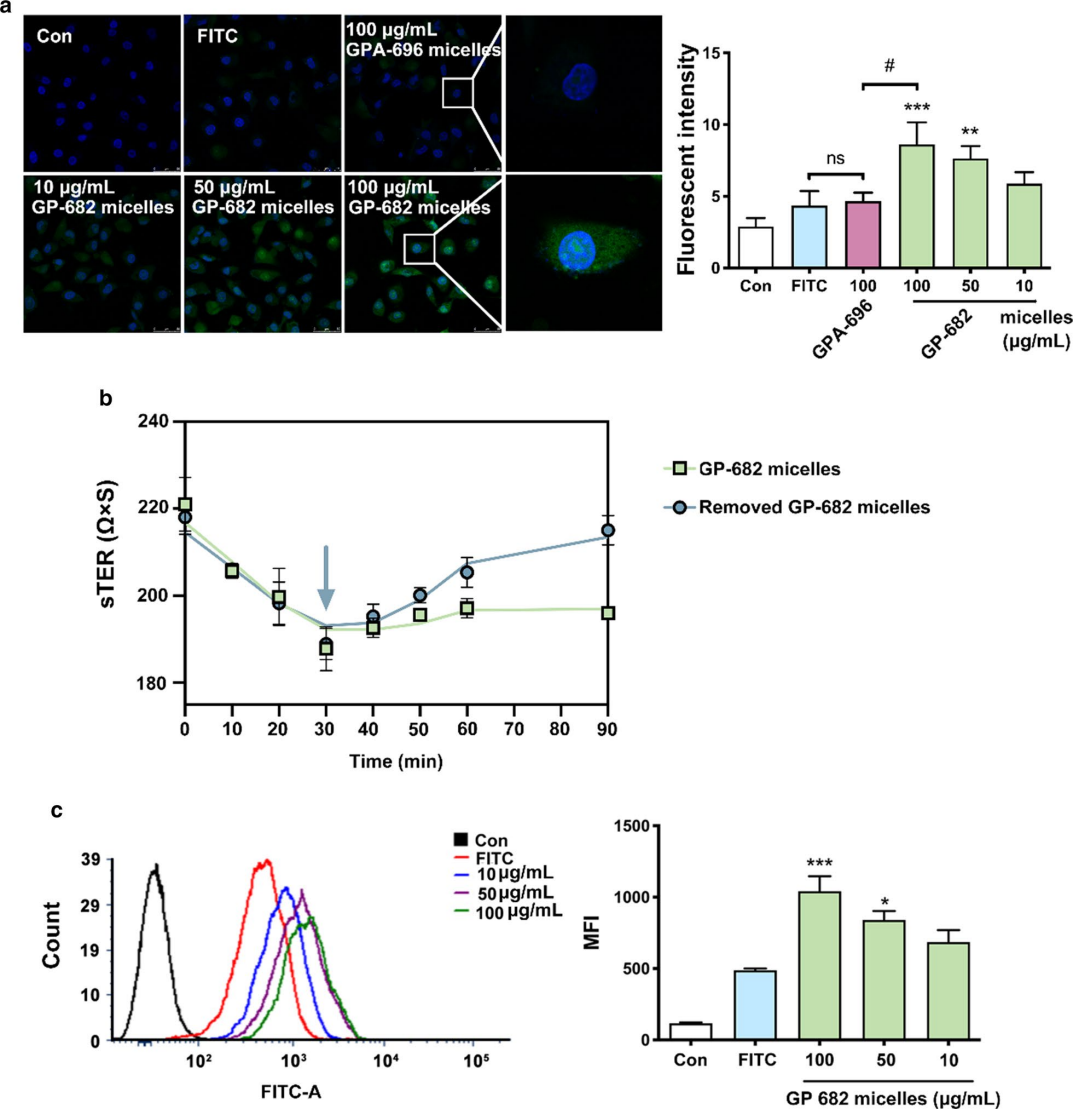
GP-682 micelles enhanced cell membrane permeability in BEAS-2B cells. **a** Fluorescence confocal images of BEAS-2B cell uptake of FITC after 30 min preincubation with different concentrations of GP-682 micelles (10, 50, 100 μg/mL) and GPA-696 micelles (100 μg/mL). **b** The TEER of BEAS-2B cells cocultured with 100 μg/mL GP-682 micelles for 90 min and TEER of BEAS-2B cells removed from micelles at 30 min. **c** Flow cytometry intensity assay of BEAS-2B cell uptake of FITC at 30 min after pretreatment with 10, 50, and 100 μg/ml GP-682 micelles (left panel). The median fluorescence intensity (MFI) for each group as summarized in a bar graph (right panel). (Data are presented as means ± SD, n = 3. a, c, *P < 0.05, **P < 0.01, ***P < 0.001 compared to the FITC group, #p < 0.05 compared to the 100 μg/mL GPA-696 micelles group)

The cell membrane is a critical component of cellular structure, and it protects the cells by separating the cytoplasm from the outside environment. To evaluate the effects of GP-682 micelles on membrane permeability, BEAS-2B cells were used for cellular uptake experiments. FITC was selected as a tracer for confocal and flow cytometry observations. As shown in Fig. 2a, after preincubation with GP-682 micelles for 30 min, the cells contained more FITC than did untreated cells, and this uptake was dose-dependent. Under the same dose conditions, the effect of GP-682 micelles was better than that of GPA-696 micelles (p < 0.05). These results indicated that GP-682 had better structural characteristics and was more suitable as a nanoparticle-based PDDS. The results observed here, that preincubation of GP-682 micelles increased cellular uptake of FITC, indicated that GP-682 micelles may improve the membrane permeability. In addition, different cell lines were used to test the selectivity of GP-682 micelles, and the results demonstrated that GP-682 micelles might improve membrane permeability in favor of lung-derived cells (Additional file 1: Fig. S18). TEER was a representative indicator to indicate the permeability of a cell monolayer [23]. TEER of BEAS-2B cells was measured after incubation with the micelles. As shown in Fig. 2b, higher TEER values indicated that the cell layer was intact before the treatments. With the addition of GP-682 micelles, the TEER of BEAS-2B cells declined gradually and reached the minimum value at 30 min, and remained at a lower level until 90 min. Therefore, the best incubation time was selected as 30 min. When the GP-682 micelles were removed, the TEER was returned to the original level. This result also demonstrated that the changes in cell monolayer permeability were reversible. The flow cytometry experiment showed similar results to the confocal experiment, in which the cells preincubated with GP-682 micelles for 30 min also contained more FITC than the untreated cells (Fig. 2c).

### GP-682 micelles induced cell membrane perforation

**Fig. 3:**
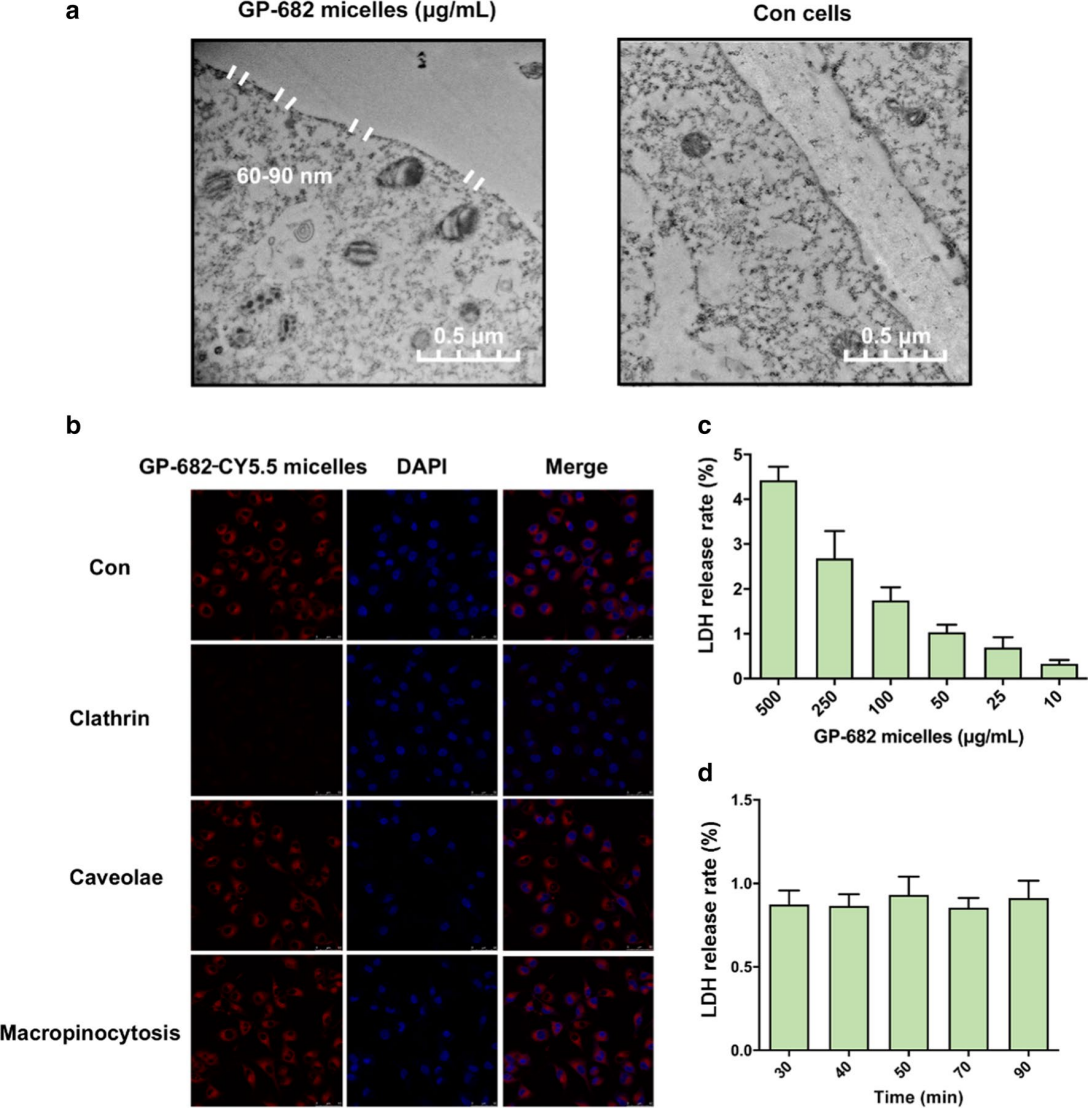
GP-682 micelles modulated cell membrane perforation and improved LDH release. **a** Morphological changes in BEAS-2B cells observed using TEM treatment with or without 100 μg/mL GP-682 micelles treatment. **b** Confocal images with GP-682 micelles in BEAS-2B cell lines showing the mechanism of cellular uptake. **c** The LDH release rate in BEAS-2B cells with 30 min treatment with 10 to 500 μg/mL GP-682 micelles. **d** The LDH release rate in BEAS-2B cells with 50 μg/mL GP-682 micelles for 30 to 90 min

To further verify the effective cell membrane permeability of the GP-682 micelles, TEM images of BEAS-2B cells were observed. As shown in Fig. 3a, the membrane surface in control cells was a dense and continuous black line, demonstrating the integrity of the cell membrane. Whole images of cells are shown in Additional file 1 (Additional file 1: Fig. S19). In the 100 μg/mL GP-682 micelles 30 min pretreated group, some pores of approximately 60–90 nm were formed on cell membranes, and the cell morphology was normal. This was different from other saponins that induce membrane lysis and lead to the leakage of organelles [24, 25]. For example, hederacolchiside A1 formed holes in MEL-5 cells larger than 1 μm and induced cell necrosis [26]. GP-682 micelles can induce cell membrane perforation, consistent with the smaller size of the GP-682 micelle itself (60–90 nm). As a universal guide for PDDS, the GP-682 micelles prepared here may fulfill a special role for drug uptake.

Endocytosis is a common mechanism underlying the cellular uptake of nanoparticles, which is implemented via various mediators such as clathrin, caveolae and micropinocytosis [27]. The uptake mechanism of GP-682 micelles was determined with different endocytosis inhibitors, such as sucrose for inhibiting clathrin-mediated endocytosis, methyl-β-cyclodextrin for caveolae and amiloride for macropinocytosis-mediated cellular uptake. The confocal images shown in Fig. 3b indicated that the formulation of membrane perforation induced by GP-682 micelles resulted from the uptake of GP-682 micelles, which was controlled by clathrin-mediated endocytosis.

LDH, a stable cytoplasmic enzyme existing in all cells, is often used as a cytosolic marker in the cellular membrane damage test [26]. When the BEAS-2B cells were exposed to various GP-682 micelles concentrations, the LDH was released from the cells in a dose-dependent manner (Fig. 3c). The leakage of high-dose LDH indicated that cells were seriously damaged. However, leakage of less than 1% is considered safe for cells [28, 29]. Incubation of cells with 50 μg/mL GP-682 micelles did not lead to an increase in LDH release as time proceeded (Fig. 3d). These results demonstrated that appropriate GP-682 micelles could change the permeability of the cell membrane by perforating cell membranes, but maintained cell integrity.

### GP-682 micelle preincubation enhanced the efficacy of cellular uptake

**Fig. 4:**
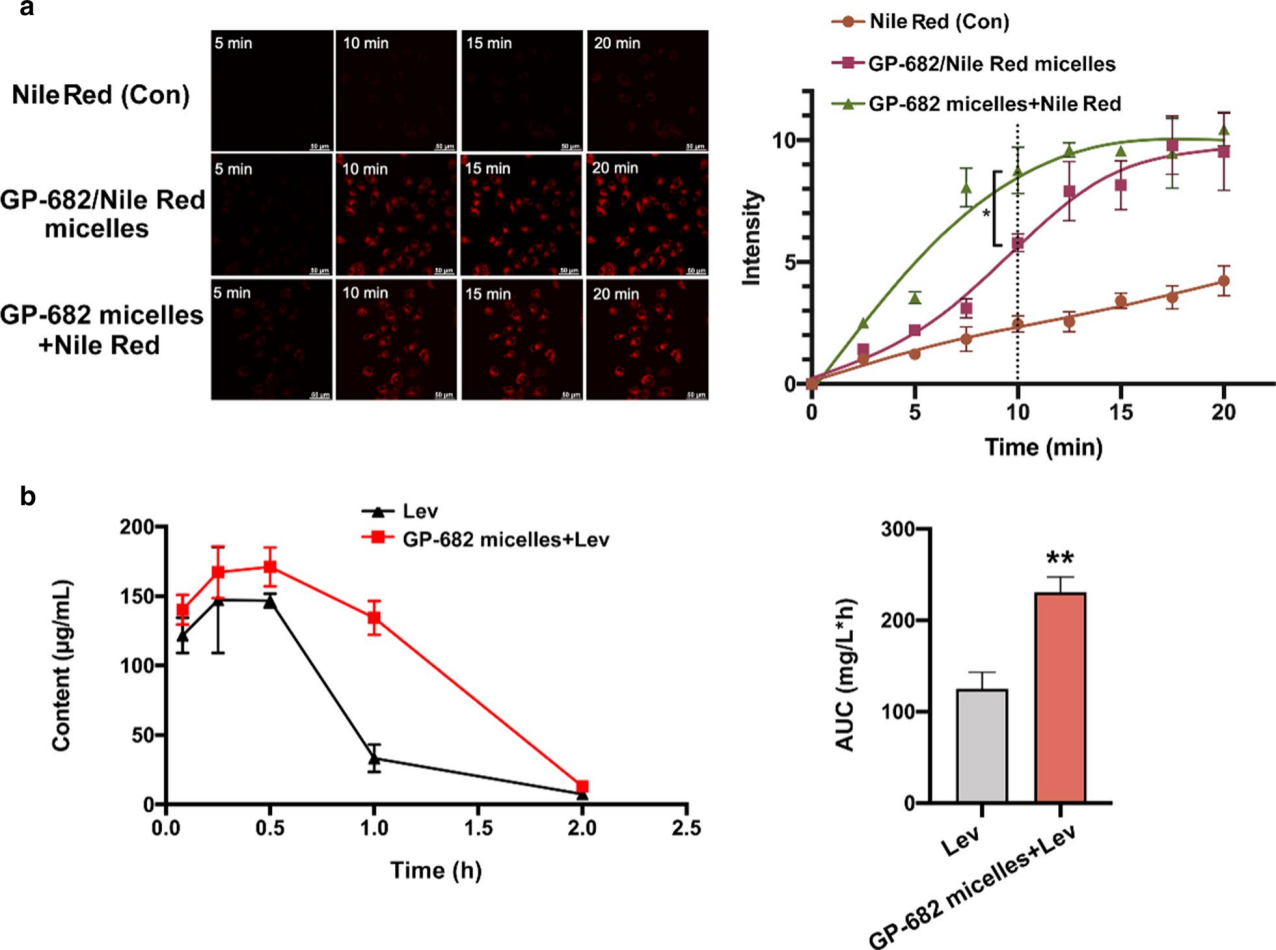
GP-682 micelles enhanced cellular uptake in vitro and improved drug lung targeting in vivo. **a** BEAS-2B cells treated with Nile Red alone, GP-682/Nile Red micelles, and Nile Red pretreated with 100 μg/mL GP-682 micelles. Fluorescence of Nile Red was observed for 20 min using a confocal microscope. (Data are presented as means ± SD, n = 3, *p < 0.05 compared to the GP-682/Nile Red micelles in 10 min). **b** Mice were preinjected with or without GP-682 micelles (80 mg/kg) for 30 min, then injected with Lev. The pharmacokinetic parameters of Lev in mouse lung tissue were quantified using HPLC. (Data are presented as means ± SD, n = 5, **p < 0.01 compared to the Lev control group)

Changes in cell membrane permeability are important indicators of drug uptake by cells [30]. Nile Red is an excellent fluorescent lipid probe that is strongly fluorescent only in a lipid environment. When Nile Red is dispersed in an aqueous solution, almost no fluorescence is observed [31]. Based on this phenomenon, it was used as a representative of the drug combinations to enable tracing in the cells. To investigate the effect of GP-682 micelles on drug entry into cells, two different delivery methods, preincubation with GP-682 micelles or drug directly carried with GP-682 micelles (GP-682/Nile Red micelles), were used to test the cellular uptake effectiveness in vitro*.* The images of Nile Red entering cells and its fluorescence statistics are shown in Fig. 4a. Cells in the control group exhibited a slow uptake rate of Nile Red within 20 min of observation. Compared with the control group, the GP-682 micelles group and GP-682/Nile Red micelles group had faster ingestion speeds. The cells pretreated with GP-682 micelles contained more red dye than the cells treated with GP-682/Nile Red micelles alone. These data suggest that preincubation with GP-682 micelles achieves higher performance than incubation with the GP-682/Nile Red micelles, which have Nile Red wrapped in hydrophobic cavities (P < 0.05). Membranes provide an amphiphilic environment, and the self-assembling structure is similar to GP-682 micelles. This structural similarity may lead to the ability to interact with the cell membrane, allowing perforation of the cell membrane for drug intake [19].

### GP-682 micelles changed the distribution of Lev in mouse lung

Generally, high doses of antibiotics contribute to side effects, such as gastrointestinal reaction, skin allergic reaction and nervous system response [32]. Therefore, a new strategy to reduce the use of high doses of antibiotics is needed. The active compound 682 increases drug targeting in mouse lung [17], and GP-682 micelles effectively improved cellular uptake in vitro*.* To evaluate whether GP-682 micelles achieve higher lung tissue uptake in vivo, the concentration of Lev in lung tissue was detected using HPLC with or without GP-682 micelle pretreatment for 30 min. As shown in Fig. 4b, the GP-682 micelles combined with Lev group displayed a higher level of Lev in lung tissue than the Lev group. The AUC_0-t_ of the GP-682 micelles combined with Lev group was 224.017 ± 34.494, and the AUC_0-t_ of the Lev group was 129.796 ± 16.161. Compared with the Lev group, the AUC_0-t_ of the GP-682 micelles combined Lev group was 1.8 times that of the Lev group. The C_max_ of Lev in the GP-682 micelles combined with Lev group was increased, and the clearance rate CLz/F was significantly decreased compared with the Lev group. The detailed pharmacokinetic parameters of Lev in mouse lung are shown in Additional file 1: Table S4. This result suggested that GP-682 micelles increased the lung delivery of Lev by improving lung cell permeability, which increased Lev retention in lung cells, and may promote the antibacterial effect in vivo.

### GP-682 micelles combination improved antibacterial effects and guaranteed survival rate

**Fig. 5:**
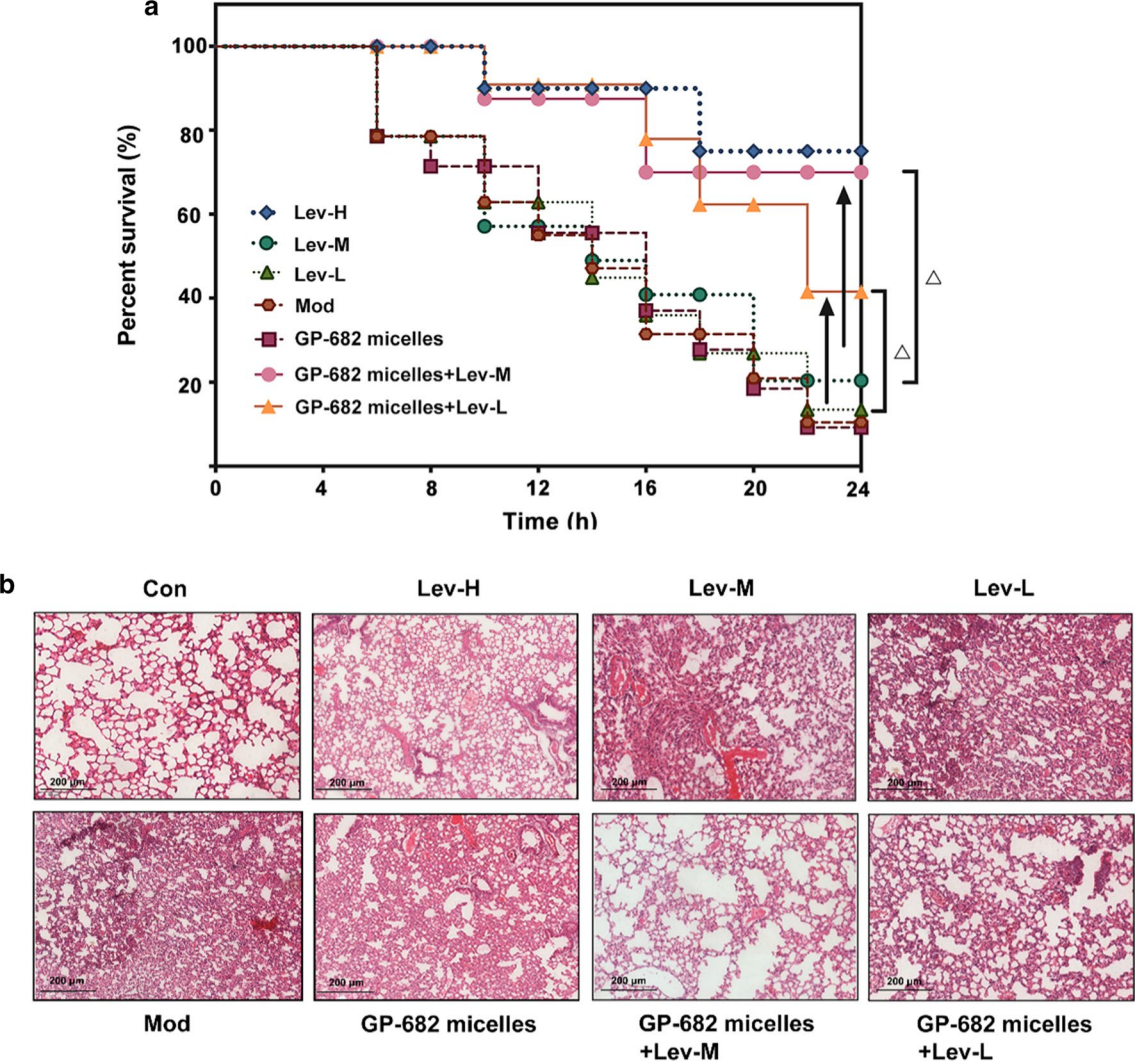
GP-682 micelles combined with Lev enhanced mouse survival rate and improved lung inflammation in mice. **a** The combined application of GP-682 micelles improved mouse survival rate. The KM mice were infected with PA 14 strains followed by injection of Lev with or without GPD-682 micelles combination. Survival of mice was recorded at 6, 8, 10, 12, 14, 16, 18, 20, 22 and 24 h, respectively; **b** H&E staining images of the mouse lungs. (Data are presented as means ± SD, n = 15, ∆p < 0.05 compared to the Lev group)

Lev is usually used as an antibiotic in treating *P. aeruginosa*-induced infections in mice [33]. To evaluate GP-682 micelles’ benefits on the adjuvant therapeutic effect, a PA 14 strain of *P. aeruginosa*-induced acute lung infection model was established in this paper. As shown in Fig. 5a, mice in the GP-682 micelles group exhibited the same median survival rate as in the model group (14%). This result demonstrated that GP-682 micelles did not have an antibacterial effect when used alone. Mice in the Lev-treated group showed a higher median survival rate than in the model group. When low and medium doses of Lev were combined with GP-682 micelles, the survival rate was markedly enhanced compared to the corresponding Lev group (p < 0.05).

**Fig. 6:**
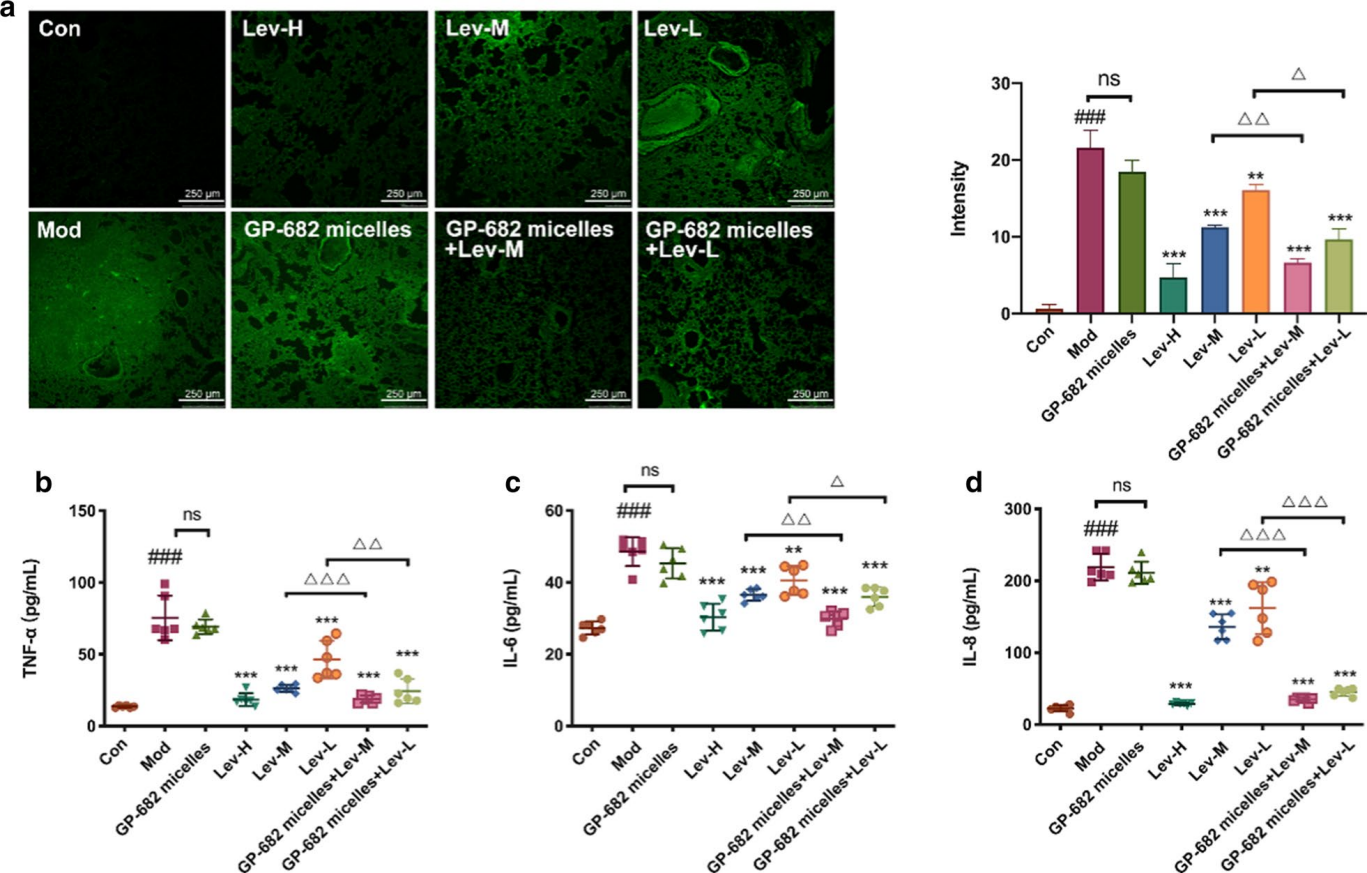
GP-682 micelles improved the antibacterial effect. **a** Immunofluorescence images of PA 14 strains distributed in the mouse lung. The scale bar represents 250 μm (left panel); the histogram presents the relative fluorescence of PA 14 strains (right panel) (n = 3). GP-682 micelles enhanced the anti-inflammatory effect of Lev and inhibited the production of TNF-α (**b**), IL-6 (**c**) and IL-8 (**d**) induced by acute infection with PA 14 strains (n = 6). (Bars represent mean ± SD, Con: control group; Mod: model group; Lev: Lev group; GP-682 micelles: GP-682 micelles group; GP-682 micelles + Lev: combined GP-682 micelles and Lev group. **p < 0.01, ***p < 0.001 compared to the Mod group; ###p < 0.001 compared to the Con group; ∆p < 0.05, ∆∆p < 0.01, ∆∆∆p < 0.001 compared to the Lev group)

The mouse lungs were observed with H&E staining to detect the anti-infective effect of Lev with or without GP-682 micelles on PA 14-induced mouse acute lung injury. As shown in Fig. 5b, the lungs of mice in the model group and the GP-682 micelles group exhibited severe bleeding, widened alveolar septa and cell infiltration compared with the control group. Pretreatment with Lev or GP-682 micelles combined with Lev protected the lung from visible damage. To further test the anti-infective effect, the distribution of PA 14 strains in mouse lung tissue was detected using immunofluorescence staining via a fluorescent-labeled Anti-*P. aeruginosa* antibody. As shown in Fig. 6a, the immunofluorescence in the model group and the GP-682 micelles group was significantly stronger than in the control group, but the Lev group showed a significant and dose-dependent decrease. The fluorescence in the GP-682 micelles combination group revealed that the low dose of Lev combined with GP-682 micelles showed the same effects as the middle dose of Lev. The middle dose of Lev combined with GP-682 micelles produced the high dose effects of Lev. This result was consistent with the results of H&E staining. Pulmonary perfusates in all groups were also collected to detect inflammation-related factors, including TNF-α, IL-6 and IL-8 induced by PA 14 strains. The results shown in Fig. 6b–d are consistent with the immunofluorescence experiment. GP-682 micelles did not generate an antibacterial effect according to these results. However, mice in the GP-682 micelles combination group had the equivalent antibacterial effect compared to the mice treated by a relatively high dose of Lev. All of these results further confirmed that GP-682 micelles could improve the distribution of Lev in the mouse lung by increasing lung permeability in mice and, thus, exerted a novel protective antibacterial effect.

Previous nanoparticle-based pulmonary drug delivery systems require encapsulating drugs into the nanomaterials in advance. For example, rifampicin, an antibiotic drug used to treat tuberculosis and other pulmonary infections, was encapsulated into porous nanoparticle-aggregate particles to achieve shelf stability and prolong the time of action of rifampicin in the lung [34]. Our GP-682 nanomicelles combined with Lev (which could also be used to combine other drugs) provided a more flexible pulmonary drug delivery channel.

## Conclusions

In summary, a natural PSS derived from Platycodi radix, GP-682, was used to design and prepare novel self-assembled nanomicelles for application as a PDDS for the first time. Preincubation of GP-682 micelles enhanced the cell membrane permeability and helped to improve drug uptake in vitro. The benefits increased the distribution of the antibiotic Lev in mouse lung tissue and presented a better anti-infection efficacy in vivo. The combination with the GP-682 micelles will provide another effective potential treatment for use in lung-targeting therapy. By explaining the mechanism of GP-682 for cell membrane permeability enhancement, a new PDDS application mode of nanomicelles was developed. Unlike most micelles used as cargo for drug delivery, the self-assembled GP-682 micelles may represent a universal technique in lung-targeting therapy.

## Supplementary information

**Additional file 1: Fig. S1.** High-resolution mass spectrum of GP-682 in positive and negative mode. **Fig. S2.** The 1H NMR data of GP-682. **Fig. S3. **The 13C NMR data of GP-682. **Fig. S4.** The heteronuclear single quantum coherence (HSQC) NMR data of GP-682. **Fig. S5. **The chemical structure of GPA-696.** Fig. S6. **High-resolution mass spectrum of platycodon 696 in positive and negative mode. **Fig. S7. **The 1H NMR data of GPA-696. **Fig. S8.** The 13C NMR data of GPA-696. **Fig. S9. **The heteronuclear single quantum coherence (HSQC) NMR data of GPA-696. **Fig. S10. **The synthetic route of alkynyl-GP-682. **Fig. S11.** The molecular mass of alkynyl-GP-682. **Fig. S12.** The size distribution of GP-682-Cy5.5 micelles. **Fig. S13. **The synthetic route of GP-682-Cy5.5 and Tyndall phenomenon of micelles. **Fig. S14.** HPLC chromatogram of Lev and terazosin hydrochloride (internal standard) in lung tissue. **Fig. S15.** Quantitative standard curve of Lev in lung tissue. **Fig. S16. **The cell viability of BEAS-2B cells treated by GP-682 micelles. **Fig. S17. **The impact of GP-682 micelles in BEAS-2B cell cycle. **Fig. S18. **Fluorescence confocal images of cell uptake of FITC after 30 min preincubation of 100 μg/mL GP-682 micelles. **Fig. S19.** The whole images of BEAS-2B cells observed by TEM treatment with or without 100 μg/mL GP-682 micelles treatment. **Table S1.** Investigation of the linear relationship of Lev in lung tissue.** Table S2.** Intra and inter-day precision test. **Table S3. **Results of recovery of Lev in lung tissue samples. **Table S4.** Pharmacokinetic parameters of Lev in mice lung.

## Data Availability

The datasets supporting the conclusions of this article are included within the article and its Additional file 1.

## Abbreviations

GP-682: 3-*O*-β-D-glucopyranosyl platycodigenin 682
PDDS: Pulmonary drug delivery system
TEM: Transmission electron microscopy
LDH: Lactate dehydrogenase
Lev: Levofloxacin
pMDI: Pressurized metered-dose inhalers
DPI: Dry powder inhalers
SLN: Solid lipid nanoparticles
NLC: Nanostructured lipid carrier
GPA-696: 3-*O*-B-d-glucopyranosylplatyconic acid 696
PSS: Platycodon secondary saponins
DLS: Dynamic light scattering
CMC: Critical micelle concentration
BEAS-2B cells: Human normal lung epithelial cells
CCK8: Cell Counting Kit-8
HPLC: High-performance liquid chromatography
H&E: Hematoxylin–eosin
