# Supplementary material for: Micelles self-assembled by 3-O-β-d-glucopyranosyl latycodigenin enhance cell membrane permeability, promote antibiotic pulmonary targeting and improve anti-infective efficacy
Source: J Nanobiotechnology. 2020 Oct 2;18:140. doi: 10.1186/s12951-020-00699-y (PMC7532624; doi:10.1186/s12951-020-00699-y)
Supplement: Supplementary file 1 — Additional file 1: Fig. S1. High-resolution mass spectrum of GP-682 in positive and negative mode. Fig. S2. The 1H NMR data of GP-682. Fig. S3. The 13C NMR data of GP-682. Fig. S4. The heteronuclear single quantum coherence (HSQC) NMR data of GP-682. Fig. S5. The chemical structure of GPA-696. Fig. S6. High-resolution mass spectrum of platycodon 696 in positive and negative mode. Fig. S7. The 1H NMR data of GPA-696. Fig. S8. The 13C NMR data of GPA-696. Fig. S9. The heteronuclear single quantum coherence (HSQC) NMR data of GPA-696. Fig. S10. The synthetic route of alkynyl-GP-682. Fig. S11. The molecular mass of alkynyl-GP-682. Fig. S12. The size distribution of GP-682-Cy5.5 micelles. Fig. S13. The synthetic route of GP-682-Cy5.5 and Tyndall phenomenon of micelles. Fig. S14. HPLC chromatogram of Lev and terazosin hydrochloride (internal standard) in lung tissue. Fig. S15. Quantitative standard curve of Lev in lung tissue. Fig. S16. The cell viability of BEAS-2B cells treated by GP-682 micelles. Fig. S17. The impact of GP-682 micelles in BEAS-2B cell cycle. Fig. S18. Fluorescence confocal images of cell uptake of FITC after 30 min preincubation of 100 μg/mL GP-682 micelles. Fig. S19. The whole images of BEAS-2B cells observed by TEM treatment with or without 100 μg/mL GP-682 micelles treatment. Table S1. Investigation of the linear relationship of Lev in lung tissue. Table S2. Intra and inter-day precision test. Table S3. Results of recovery of Lev in lung tissue samples. Table S4. Pharmacokinetic parameters of Lev in mice lung. [file 12951_2020_699_MOESM1_ESM.docx]

**Additional file 1**

**Micelles self-assembled by 3-O-β-D-glucopyranosyl latycodigenin enhance cell membrane permeability, promote antibiotic pulmonary targeting and improve anti-infective efficacy**

Man Zhang^1^, Lili Ye^1^, Hao Huang^2^, Dandan Cheng^3^, Kaixin Liu^1^, Wenbo Wu^1^, Fukui Shen^1^, Zhihong Jiang^2^, Yuanyuan Hou^1^*, Gang Bai^1^*

E-mail address: houyy@nankai.edu.cn (Yuanyuan Hou), gangbai@nankai.edu.cn (Gang Bai)

**1. The isolation and purification of GP-682 and GPA-696**

**1.1. General Information**

High-resolution mass spectra (HRMS) were obtained with a FTICR-MS (Ion spec 7.0T) spectrometer. ^1^H NMR spectra were obtained by using a Bruker AV 400. Chemical shifts are reported in parts per million (ppm) relative to either a tetramethylsilane internal standard or solvent signals. Data are reported as follows: chemical shift, multiplicity (s = singlet, d = doublet, t = triplet, q = quartet, br = broad, m = multiplet), coupling constants and integration. ^13^C NMR spectra were recorded using a Bruker AV 400 spectrometer (100 MHz) using DMSO-*d*6 as the solvent. Chemical shifts (*δ*) are reported in parts per million measured relative to the solvent peak.

**1.2. Spectrum Data**


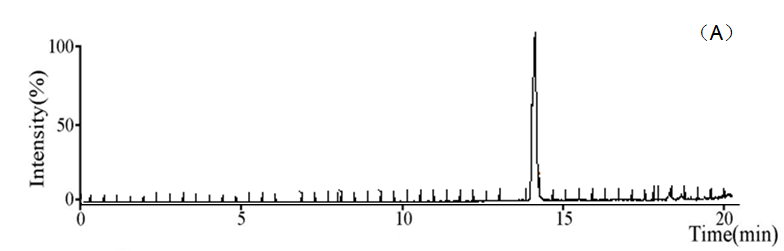


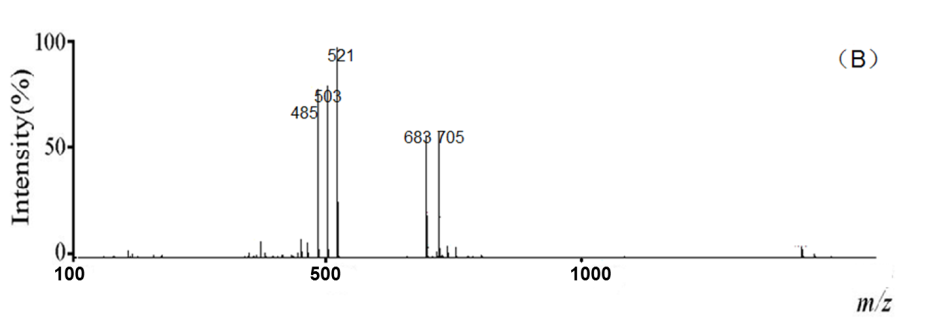


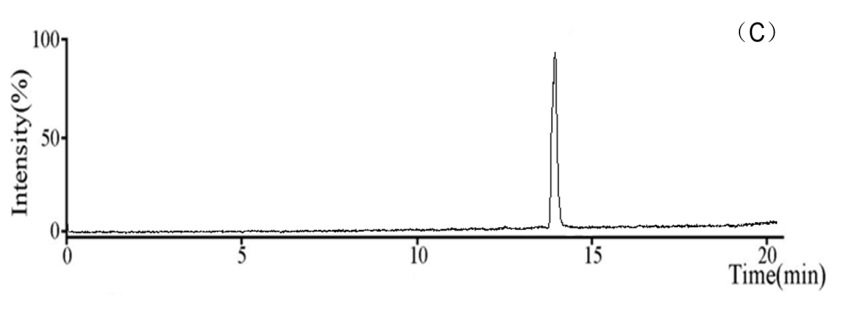


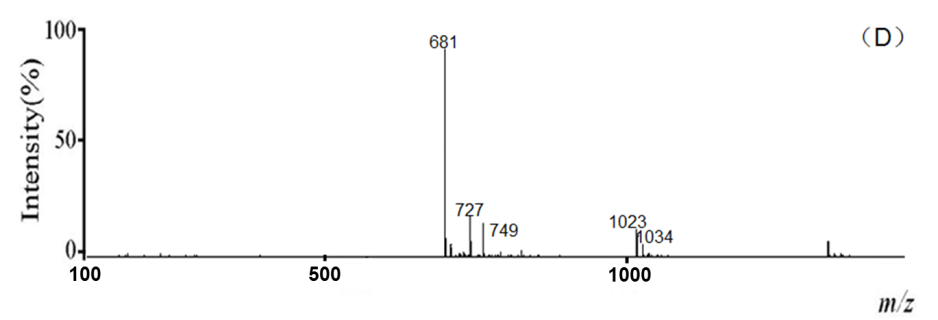


Fig. S1. High-resolution mass spectrum of GP-682 in positive and negative mode

(A), (B): positive mode (C), (D): negative mode

The NMR and HRMS of GP-682 were: ^1^H NMR (400 MHz, DMSO-*d*6) δ 5.48 (s, 1H), 5.22 (d, J = 3.9 Hz, 1H), 4.97 (s, 2H), 4.71 (d, J = 4.5 Hz, 1H), 4.53 (dt, J = 18.4, 5.1 Hz, 2H), 4.32 (s, 1H), 4.23 (d, J = 7.7 Hz, 1H), 4.11 (s, 1H), 4.05-3.96 (m, 3H), 3.80-3.61 (m, 3H), 3.49-3.36 (m, 3H), 3.15 (d, J = 13.3 Hz, 4H), 3.05 (d, J = 8.0 Hz, 2H), 2.88 (dd, J = 14.3, 4.5 Hz, 1H), 2.21 (t, J = 13.4 Hz, 1H), 1.84 (tdd, J = 28.1, 10.0, 5.9 Hz, 4H), 1.72-1.45 (m, 5H), 1.31 (s, 6H), 1.17 (d, J = 15.8 Hz, 5H), 1.09-1.03 (m, 1H), 0.96 (dd, J = 12.4, 4.4 Hz, 1H), 0.90 (s, 3H), 0.83 (s, 3H), 0.67 (s, 3H); ^13^C NMR (100 MHz, DMSO-*d*6) δ 178.2, 144.0, 121.5, 104.3, 76.9, 76.8, 73.8, 73.0, 70.2, 68.3, 61.6, 61.1, 48.6, 47.4, 46.7, 46.5, 46.4, 44.2, 41.2, 36.3, 35.2, 34.6, 32.9, 32.7, 31.5, 30.3, 26.5, 24.2, 23.1, 18.2, 17.2, 16.8. HRMS [M - H]^-^ calculated 681.3856, found 681.3853.

Fig. S2. The ^1^H NMR data of GP-682.

Fig. S3. The ^13^C NMR data of GP-682.

Fig. S4. The heteronuclear single quantum coherence (HSQC) NMR data of GP-682.


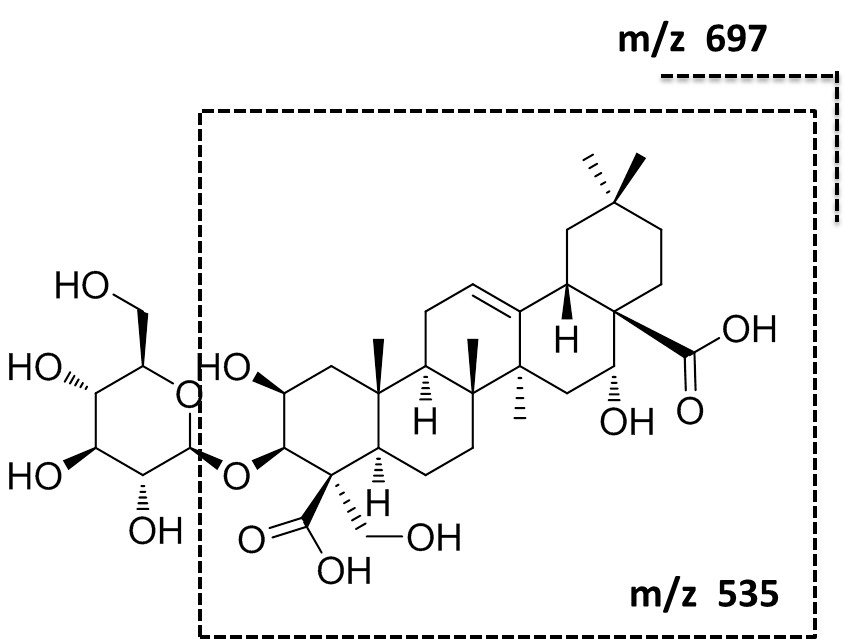


Fig. S5. The chemical structure of GPA-696.


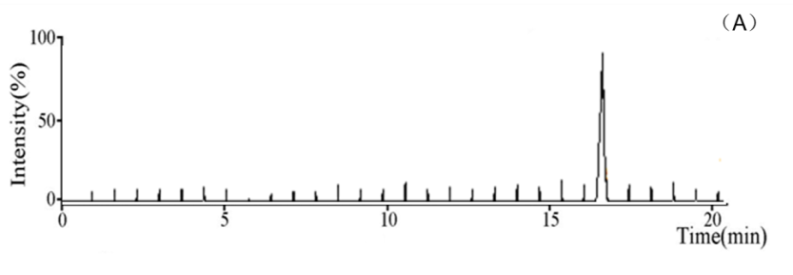

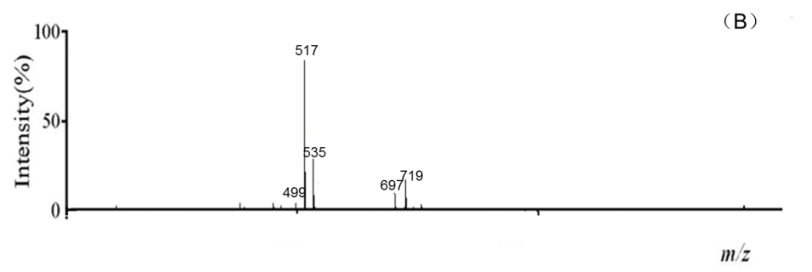

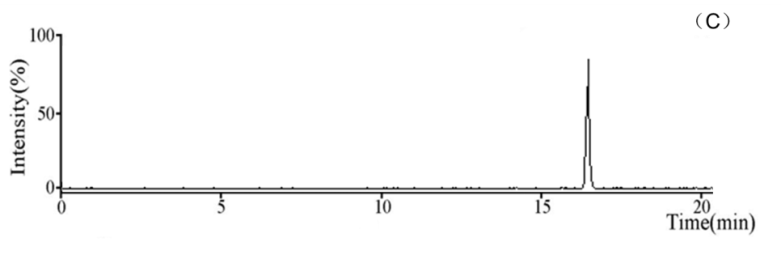

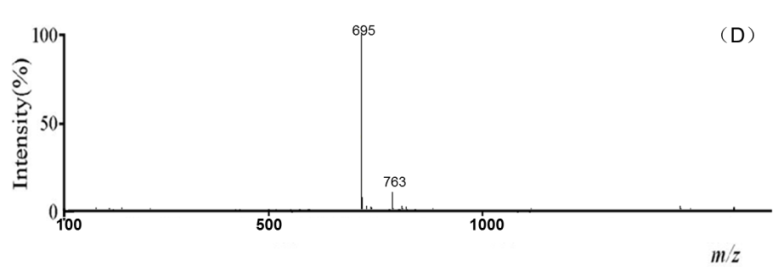


Fig. S6. High-resolution mass spectrum of platycodon 696 in positive and negative mode (A), (B): positive mode (C), (D): negative mode

The NMR and HRMS of GPA-696 were: ^1^H NMR (400 MHz, DMSO-*d*6) δ 5.21 (d, J = 3.7 Hz, 2H), 5.04 (s, 2H)4.76 (d, J = 18.1 Hz, 1H), 4.33 (s, 2H), 4.21 (s, 1H), 3.79 - 3.61 (m, 4H), 3.43 (dd, J = 11.4, 5.2 Hz, 5H), 3.16 (d, J = 7.8 Hz, 2H), 3.04 (pd, J = 8.9, 3.7 Hz, 3H), 2.88 (dd, J = 14.1, 4.5 Hz, 1H), 2.21 (t, J = 13.5 Hz, 1H), 1.81 (d, J = 32.0 Hz, 5H), 1.73-1.54 (m, 3H), 1.44 (dd, J = 23.7, 9.0 Hz, 3H), 1.31 (d, J = 6.8 Hz, 4H), 1.23 (s, 1H), 1.21-1.11 (m, 2H), 1.06 (d, J = 9.0 Hz, 4H), 0.90 (s, 4H), 0.83 (s, 3H), 0.67 (s, 3H); ^13^C NMR (100 MHz, DMSO-*d*6) δ 178.3, 144.2, 121.3, 104.4, 76.9, 76.8, 73.8, 72.9, 70.1, 68.3, 61.1, 55.1, 48.6, 47.7, 47.3, 46.4, 46.3, 41.2, 35.9, 35.2, 34.6, 32.9, 32.3, 31.5, 30.3, 26.5, 25.5, 24.2, 23.3, 16.9, 14.9. HRMS [M - H]^-^ calculated 695.3648, found 695.3651.

Fig. S7. The ^1^H NMR data of GPA-696.

Fig. S8. The ^13^C NMR data of GPA-696.

Fig. S9. The heteronuclear single quantum coherence (HSQC) NMR data of GPA-696.

**2. synthesis of GP-682-Cy5.5 and preparation of GP-682-Cy5.5 micelles**

**2.1 synthesis of alkynyl-GP-682**

GP-682 (30mg, 1eq) was dissolved in acetone solution and protected with nitrogen. The mixture of 1.1eq EDCI and 1.1eq HOBT was added and stirred for 30min. Then, 1.2eq of 3-aminopropargyne was added dropwise and stirred at room temperature for 6 h. Then the solution was extracted and concentrated with ethyl acetate for subsequent experiments. LC-MS (ESI): m/z [M+H] calculated for alkynyl-GP-682:742.4137; found: 742.4141.

GP-682 alkynyl-GP-682

Fig. S10. The synthetic route of alkynyl-GP-682.


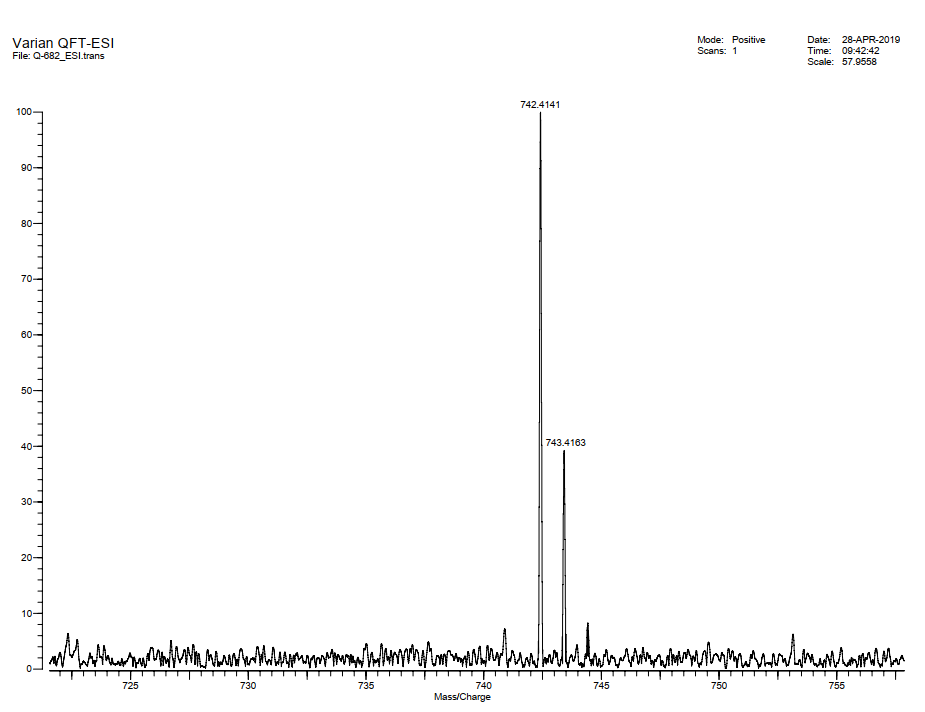


Fig. S11. The molecular mass of alkynyl-GP-682.

**2.2 synthesis of GP-682-Cy5.5**

Alkynyl-GP--682 (10mg, 1eq) was dissolved in methanol: water = 1:1. 1 eq Cy5.5 and 1 eq cuprous iodide were added in it. The reaction was stirred for 6 h, and then concentrated with ethyl acetate for subsequent experiments.

**2.3 Preparation of GP-682-Cy5.5**

The GP-682-Cy5.5 micelles were prepared using the same ultrasound method as GP-682 micelle preparation. The size distribution of GP-682-Cy5.5 micelles was detected by DLS, which was shown in Fig. S 12 the average size of GP-682-Cy5.5 micelles is 200 nm. In older to prove the successful preparation of GP-682-Cy5.5 micelles, we compared the tyndall phenomenon of GP-682-Cy5.5 micelles, GP-682/Cy5.5 micelles (Cy5.5 wrapped by GP-682, the ratio is: 1:1) and Cy5.5, in which experiment the fluorescence intensity of Cy5.5 is coincident. The excitation wavelength was 675 nm, and the emission wavelength was 700 nm. The results shown in Fig. S13 revealed that GP-682-Cy5.5 micelles had stronger tyndall phenomenon compared to the other solution, which indicated the correctness of GP-682-Cy5.5 micelles.





Fig. S12. The size distribution of GP-682-Cy5.5 micelles.


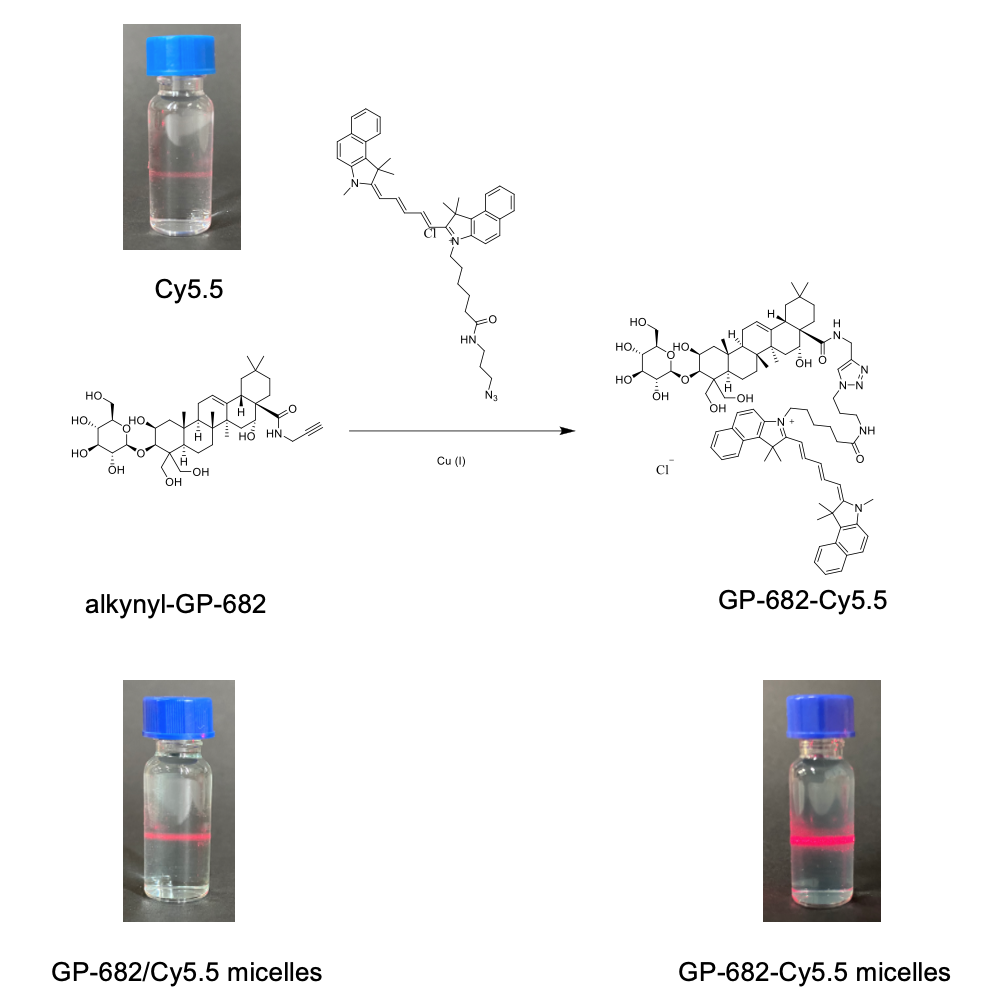


Fig. S13. The synthetic route of GP-682-Cy5.5 and Tyndall phenomenon of micelles.

**3. The methodological investigation of Lev distribution analysis of lung tissue by HPLC**

**3.1 Methods**

**3.1.1 Specificity**

The blank biological samples of mice and the biological samples after administration were taken respectively. The samples were processed according to the method described in the manuscript and determined according to the chromatographic conditions in the manuscript to investigate whether the endogenous substances in mice would interfere with the determination.

**3.1.2 Standard curve and linear range**

The reference stock solution was gradually diluted with methanol to prepare a series of standard solutions with mass concentrations of 110.7, 55.35, 27.65, 13.84, 6.92, 3.36, 1.73, 0.87, 0.43 and 0.22 μg/mL. The samples were prepared and processed according to the method described in the manuscript and determined according to the chromatographic conditions in the manuscript. Linear regression analysis was carried out with the concentration of Lev as the abscissa and the ratio of each compound to the area of internal standard peak as the ordinate.

**3.1.3 Precision and accuracy**

Took 100 μL of blank biological sample, added Lev standard solution, and prepared quality control samples of high, medium and low concentrations according to the method in the manuscript. The concentrations of quality control samples of lung tissue were 0.069, 0.554 and 4.428 μg/mL respectively, and each concentration was parallel to 5 samples. The relative error (RE) and relative standard deviation (RSD) were calculated according to the chromatographic conditions in the manuscript. The samples were prepared according to the intraday precision inspection method, and the intraday precision was investigated for 3 consecutive days.

**3.1.4 Recovery rate**

Took 100 μL of blank biological sample, added Lev standard solution, and prepared quality control samples of high, medium and low concentrations according to 3.3 "precision and accuracy", 5 samples in parallel for each concentration. Determined according to the chromatographic conditions described in the manuscript. Recorded the peak area A1, prepared another sample of Lev standard solution of the same mass concentration, determined according to the chromatographic conditions in the manuscript, recorded the peak area A2, and calculate the recovery rate with A1/A2.

**3.2 Results**

**3.2.1 Specificity**

The chromatographic peak in each biological sample has a good peak type, and there is no interference of heteropeak in the determination. The endogenous substances in the sample do not interfere with the detection results. The HPLC determination results are shown in Fig. S10.


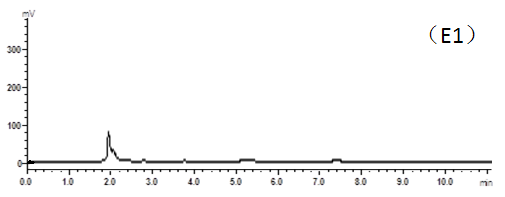

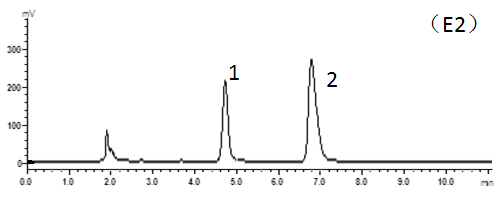


A B

Fig. S14. HPLC chromatogram of Lev and terazosin hydrochloride (internal standard) in lung tissue. A: blank lung tissue sample B: administration of lung tissue sample; 1- Lev; 2-terazosin hydrochloride.

**3.2.2 Standard curve and linear range**

In different biological samples, the lowest concentration point of the standard curve meets the requirements of S/N > 10 and RSD < 20%. The correlation coefficient of the standard curve was greater than 0.9990. The standard curve of plasma and tissue samples was shown in Fig. S11. The regression equation and linear range were shown in Table S1.


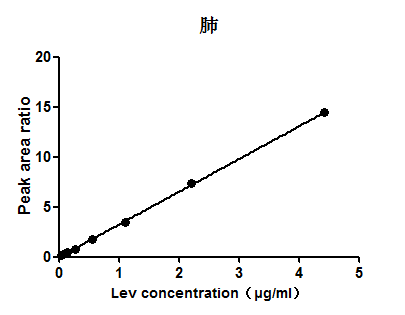


Fig. S15. Quantitative standard curve of Lev in lung tissue.

Table S1 Investigation of linear relationship of Lev in lung tissue

| samples | regression equation | correlation coefficient | linear range (μg/mL) |
| --- | --- | --- | --- |
| lung | Y=3.1331X+0.0173 | 0.9991 | 0.035～4.428 |

**3.2.3 Precision and accuracy**

The precision and accuracy were inspected. The precision in day and in day were all in accordance with RSD < 15%, Re < 15%. The inspection results were in line with the biological sample test standards.

Table S2 Intra and inter-day precision test ($\bar{x}$± *s*, n = 5)

| tissue | theoretical concentration (μg/mL) | intraday precision | | | intra-day precision | | |
| --- | --- | --- | --- | --- | --- | --- | --- |
|  |  | measured value (μg/mL) | RSD% | RE% | measured value (μg/mL) | RSD% | RE% |
| lung | 0.554 | 0.606±0.029 | 4.89 | 9.43 | 0.549±0.055 | 10.11 | -0.85 |
|  | 4.428 | 4.241±0.347 | 8.18 | -4.23 | 4.332±0.441 | 10.17 | -2.16 |
|  | 0.035 | 0.032±0.002 | 6.14 | -7.74 | 0.031±0.003 | 8.20 | -11.7 |

**3.2.4 Recovery rate**

The recovery rate of the sample was investigated. Under the treatment method of this experiment, the recovery rate was between 80% and 120%, and the inspection results meet the biological sample detection standard.

Table S3 Results of recovery of Lev in lung tissue samples ($\bar{x}$± *s*, n = 5)

| tissue | concentration added (μg/mL) | recovery rate % | RSD% |
| --- | --- | --- | --- |
| lung | 0.554 | 88.39±3.15 | 3.57 |
| lung | 4.428 | 97.12±2.51 | 2.58 |
| lung | 0.035 | 101.92±7.76 | 7.62 |

**4. Cytotoxicity test**

BEAS-2B cells were seeded in 96-well plates and incubated for 12 h, then GP-682 micelles (1.56 to 200 μg/mL) were added to cells for 24 h at 37°C. 20 μL CCK8 (HY-K0301, MedChemExpress co. td., America) solution was added to each well to co-culture with cells at 37°C for 1 h. The optical density (OD) value at 450 nm was measured by a BioTek Elx800 microplate reader (BioTek Instruments, Inc. USA). As a result, BEAS-2B cells co-cultured with GP-682 micelles for 24 h maintained a healthy state, which was detected by the CCK8 cytotoxicity test.

**
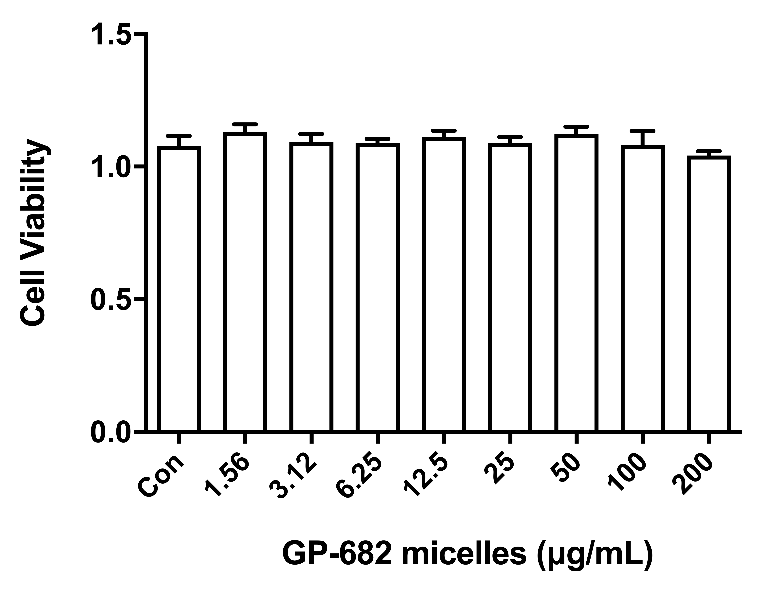
**

Fig. S16. The cell viability of BEAS-2B cells treated by GP-682 micelles. (n=6)

**5. The cell cycle detection of BEAS-2B cells**

BEAS-2B cells were seeded in a six-well culture plate (cell density, 1 × 10^5^ cells/well) and were incubated for 24 h. Next, the cells were incubated with the indicated concentrations of GP-682 micelles (50-500 μg/mL) for 24 h. For FACS analysis, the cells were detached using trypsin and were fixed overnight in ice-cold 70% ethanol. After fixation, the cells were treated with RNase A (100 μg/mL) for 30 min at 37°C and stained with PI (10 μg/mL) for 30 min at 4°C. The percentages of cells in the G0/G1, G2/M and S phases of the cell cycle were analyzed using Modfit software. As shown in Fig S17, only 500 μg/mL GP-682 micelles increased the population of cells in the G2/M phase slightly. This result demonstrated the safety limit of GP-682 micelles used in this research, which maximum concentration was 200 μg/mL.


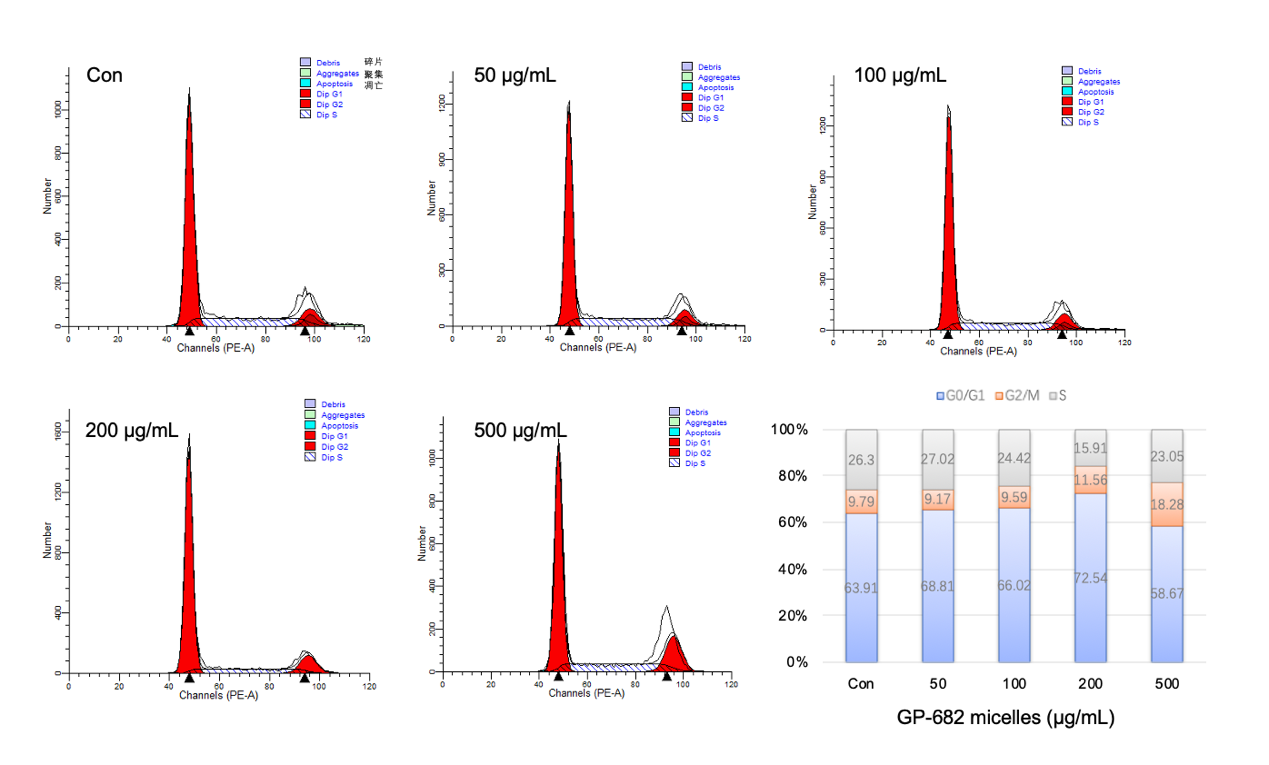


Fig. S17. The impact of GP-682 micelles in BEAS-2B cell cycle.

**6. The lung selectivity of GP-682 micelles in cell lines**

Seven cell lines including BEAS-2B cells, H9C2 cells, HepG2 cells, HK-2 cells, ECRTA cells (Endothelial Cells of Rat Thoracic Aorta), A549 cells and H295R cells were used to detect the selectivity of GP-682 micelles. Cells were cultured in small confocal dishes (NEST, 801001). 100 μg/mL GP-682 micelles were co-cultured with cells for 30 min in the culture medium at 37°C. Then the FITC (1×10-^6^mol/L) was added to cells for 10 min at 37°C. After washing by precooled PBS, the cells were fixed with 4% paraformaldehyde. A confocal microscope (Leica TCS SP8) was used to investigate the entry of FITC into the cells. The excitation wavelength was 488 nm, and the emission wavelength was 600 nm to 670 nm. The fluorescence intensity of FITC was detected by ImageJ software. As shown in Fig. S18, lung cells took in more FITC compared to other cells after incubated by GP-682 micelles, demonstrated the GP-682 micelles might improve membrane permeability in favor of lung-derived cells.


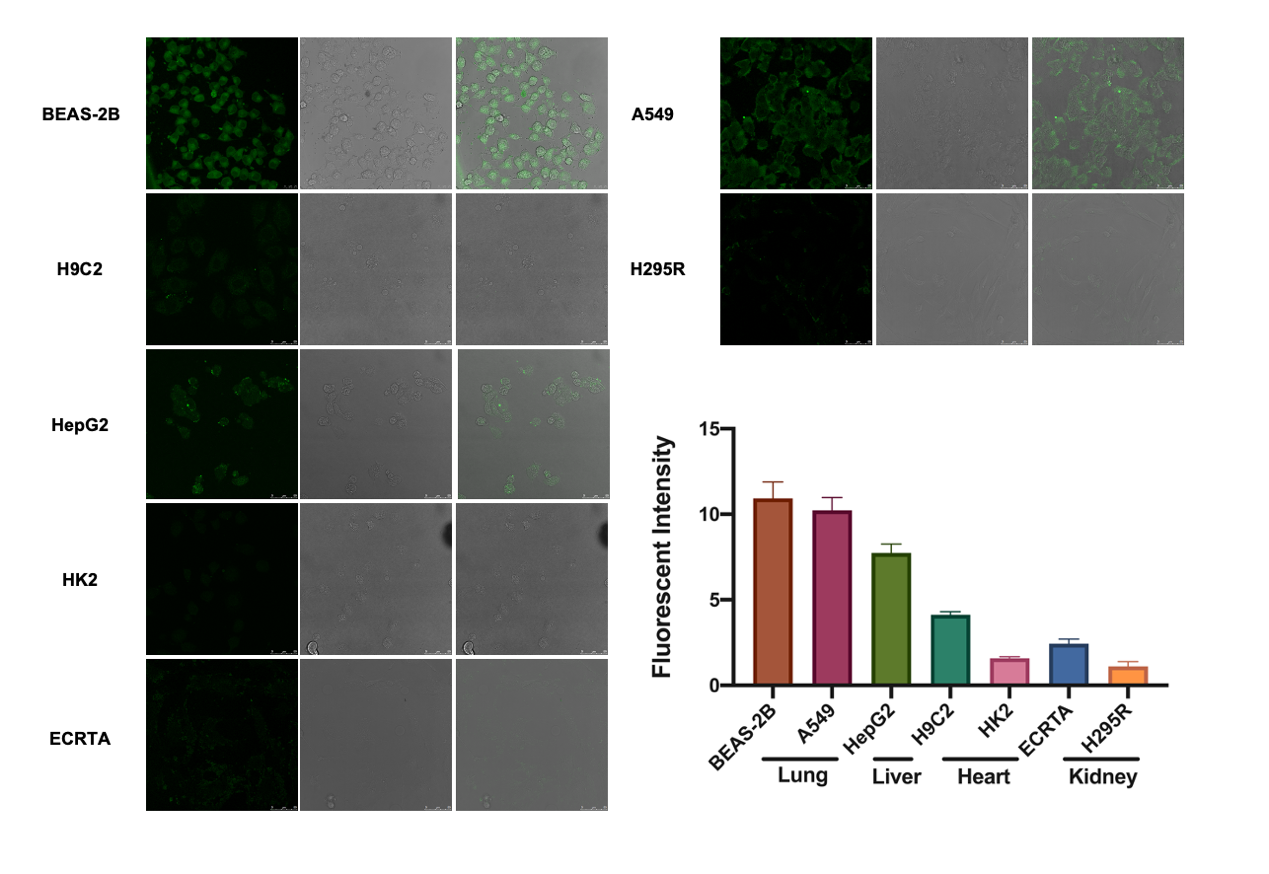


Fig. S18. Fluorescence confocal images of cell uptake of FITC after 30 min preincubation of 100 μg/mL GP-682 micelles.

**7. GP-682 micelles induced cell membrane perforation**

**
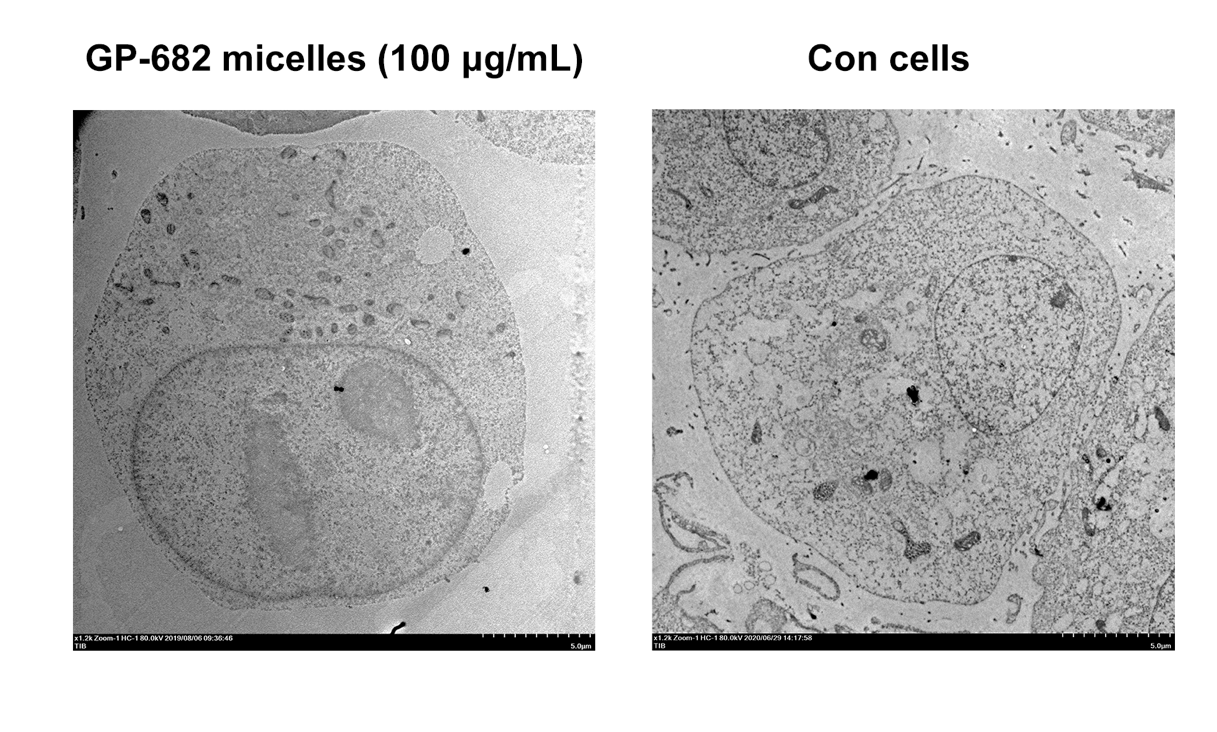
**

Fig. S19. The whole images of BEAS-2B cells observed by TEM treatment with or without 100 μg/mL GP-682 micelles treatment.

**8. Pharmacokinetic investigation of Lev in mice lung**

Table S4 Pharmacokinetic parameters of Lev in mice lung ($\bar{x}$±*s* n=5)

| parameter | unit | Lev group | GP-682 micelles  + Lev group |
| --- | --- | --- | --- |
| AUC_0-t_ | mg/L*h | 129.796 ± 16.161 | 224.017 ± 34.494 |
| C_max_ | mg/L | 178.394 ± 39.339 | 187.191 ± 17.069 |
| T_1/2Z_ | h | 0.356 ± 0.035 | 0.457 ± 0.051 |
| T_max_ | h | 0.266 ± 0.15 | 0.316 ± 0.182 |
| Vz/F | L/kg | 0.312 ± 0.053 | 0.232 ± 0.053 |
| CLz/F | L/h/kg | 0.606 ± 0.076 | 0.35 ± 0.053 |

Pharmacokinetic parameters were analyzed by DAS 2.0 software.
